# Selective separation of planar and non-planar hydrocarbons using an aqueous Pd_6_ interlocked cage†

**DOI:** 10.1039/d2sc04660a

**Published:** 2022-09-14

**Authors:** Debsena Chakraborty, Rupak Saha, Jack K. Clegg, Partha Sarathi Mukherjee

**Affiliations:** Department of Inorganic and Physical Chemistry, Indian Institute of Science Bangalore 560012 India psm@iisc.ac.in; School of Chemistry and Molecular Biosciences, The University of Queensland St. Lucia Queensland 4072 Australia

## Abstract

Polycyclic aromatic hydrocarbons (PAHs) find multiple applications ranging from fabric dyes to optoelectronic materials. Hydrogenation of PAHs is often employed for their purification or derivatization. However, separation of PAHs from their hydrogenated analogues is challenging because of their similar physical properties. An example of such is the separation of 9,10-dihydroanthracene from phenanthrene/anthracene which requires fractional distillation at high temperature (∼340 °C) to obtain pure anthracene/phenanthrene in coal industry. Herein we demonstrate a new approach for this separation at room temperature using a water-soluble interlocked cage (1) as extracting agent by host–guest chemistry. The cage was obtained by self-assembly of a triimidazole donor L·HNO_3_ with *cis*-[(tmeda)Pd(NO_3_)_2_] (M) [tmeda = *N*,*N*,*N*′,*N*′-tetramethylethane-1,2-diamine]. 1 has a triply interlocked structure with an inner cavity capable of selectively binding planar aromatic guests.

## Abstract 2

We report here a triply interlocked cage with the ability to encapsulate planar guests in aqueous medium. This property was then employed to efficiently separate planar and non-planar aromatic hydrocarbons by aqueous extraction.

## Graphical abstract

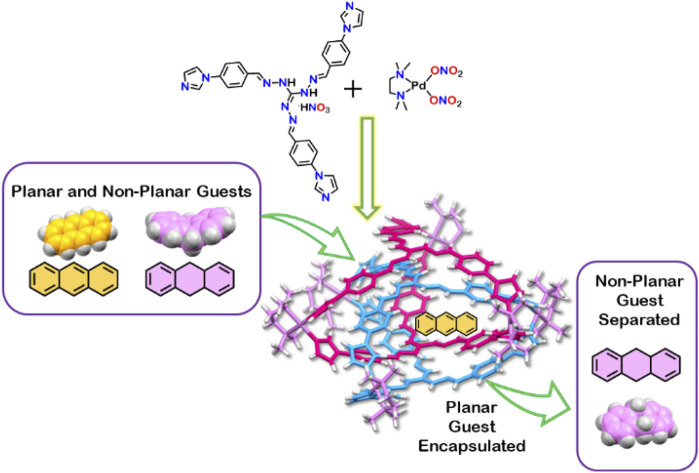

## Introduction

Selective sequestration of substrates is a crucial step for many enzymatic reactions and biological processes.^1^ In such processes, enzymes or other proteins show highly specific binding of guests within suitable pockets using mechanisms such as Fischer's lock and key^2^ or Koshland's induced fit.^3^ Inspired by such systems, multiple artificial analogues have been designed to mimic the binding property of naturally occurring host molecules.^4^ These artificial analogues can be broadly classified into macromolecular hosts and supramolecular or molecular hosts.^5^ Macromolecular hosts include porous covalent and coordination frameworks which often have very low solubility in common solvents. This leads to a loss of solution-processability and restricts their uses.^5^ Other molecular hosts, such as porous organic cages,^6^ mostly exhibit selective guest uptake in their solid or crystalline state. This limits their application to uptake of only gases and low-boiling liquids, and therefore, selective uptake between solid substrates is rarely achieved.^7^ Supramolecular hosts are formed by the self-assembly of multiple subunits. Such hosts, like the ones formed by metal–ligand coordination,^8–11^ have multiple advantages such as synthetic ease and solubility in common solvents, including water. Specifically, water-soluble hosts have a high affinity for guest encapsulation due to multiple hydrophobic interactions.^12^ Owing to these advantages, self-assembled coordination systems have been used for multiple applications ranging from light-harvesting,^13^ catalysis,^14^ sensing,^15^ separation,^16^ and stabilization of reactive intermediates.^17^

Polycyclic aromatic hydrocarbons (PAH) are common planar compounds which are ideal candidates for fabricating electronic and optical devices because of their rigid, planar, and conjugated structure.^18^ Although important, such molecules are micropollutants in water^18^ and hence their segregation in water is environmentally beneficial.^16,19^ The hydrogenation of such PAHs is often employed for making derivatives to tune the optoelectronic properties of these molecules. However, such hydrogenated products have similar physical properties as their parent PAHs. For example, benzene and cyclohexane have similar boiling points of *ca.* 80 °C; naphthalene and 1,2,3,4-tetrahydronaphthalene have boiling points of 218 °C and 207 °C, respectively, and thus cannot be separated by simple distillation. Similarly, anthracene (A) obtained naturally in coal tar is mixed with its isomer phenanthrene (P), and carbazole. To obtain pure anthracene from this crude, first hydrogenation is carried out to predominantly form 9,10-dihydroanthracene (H_2_A) and unreacted phenanthrene and carbazole. Carbazole can be easily separated from the mixture because of its difference in solubility. However, to separate phenanthrene and 9,10-dihydroanthracene, high temperature (above 340 °C) fractional distillation is required, followed by recrystallization to get highly pure 9,10-dihydroanthracene which is then aromatized to obtain pure anthracene.^20^ This process is highly energy-intensive and inefficient. This same separation is further complicated by the fact that 9,10-dihydroanthracene and anthracene have identical *R*_f_ values in most solvents and thus chromatographic techniques are not useful specially in the milligram to gram scale range. Thus, an alternate strategy for the separation of 9,10-dihydroanthracene from its planar analogue anthracene and its isomer phenanthrene is highly desirable in coal industries.

To achieve such selective separation through host–guest chemistry, a host is required which can selectively encapsulate planar hydrocarbons. Many synthetic water-soluble host molecules are unsuitable for the selective encapsulation of planar hydrocarbons due to their large size and shape.^7,16,21^ Therefore, we shifted our interest towards molecular interlocked materials (MIMs) as host molecules. These compounds are formed by the interlocking or intertwining of molecular fragments so that the fragments cannot be separated from one another without breaking the whole molecule.^22^ However, because of the interpenetrated nature of such structures, they are often incapable of guest encapsulation.^22^ Furthermore, many metal–ligand coordination based interlocked systems are formed through host–guest interaction^23^ or by templation^24^ of molecular fragments and are stable only in the presence of such guests.^24^ Thus, these systems do not exhibit any active cavity for the uptake and release of guest molecules. Probably, it is because of this reason that most interlocked cages are revered for their difficulty in synthesis and aesthetic beauty, but their application is still underexplored.

**Scheme 1:**
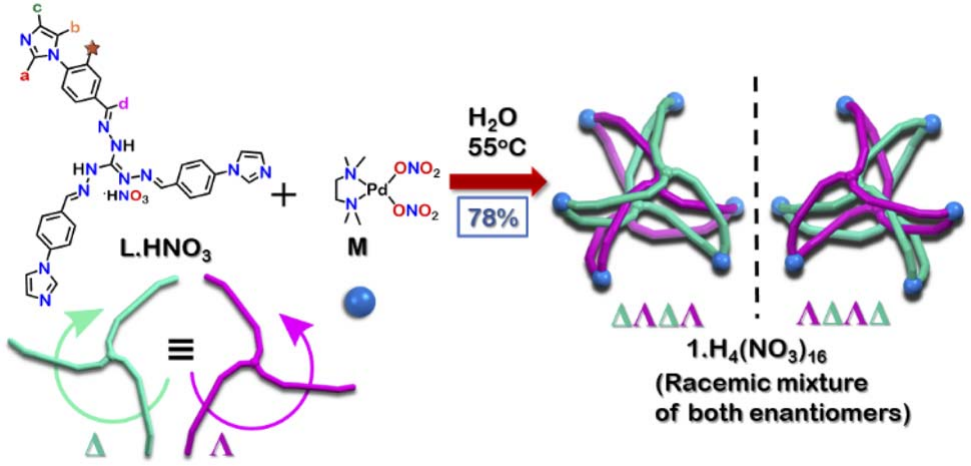
Synthesis of interlocked Pd_6_ cage 1 using a triimidazole donor L·HNO_3_.

Herein, we report the synthesis of a water-soluble triply interlocked cage 1, formed by metal–ligand coordination-driven self-assembly of guanidine-based triimidazole ligand L·HNO_3_ with a *cis*-blocked Pd(ii) acceptor M (Scheme 1). Owing to the shape of the guanidine core, interlocked cage 1 had an inner cavity with dimensions suitable for guest uptake. 1 facilitated the uptake and release of planar guest molecules without disrupting the interlocked structure. Further investigations revealed that 1 showed selective uptake of planar aromatic guests over their hydrogenated non-planar analogues. Through this, efficient separation of 9,10-dihydroanthracene (H_2_A) from anthracene (A) and phenanthrene (P) could also be achieved. To the best of our knowledge, the present results demonstrate for the first time an example of a water-soluble interlocked molecular host capable of showing guest encapsulation and de-encapsulation, and challenging separation of phenanthrene/anthracene from non-planar 9,10-dihydroanthracene by simple aqueous extraction at ambient conditions.

## Results and discussion

### Synthesis and characterization

The tri-imidazole ligand L·HNO_3_ was synthesized by the condensation of 4-(1*H*-imidazol-1-yl)benzaldehyde and triaminoguanidinium nitrate in a ethanol/water (3 : 1) mixture in good yield (Scheme S1†).^25^ The product was characterized by ESI-MS in methanol and multinuclear NMR in DMSO-*d*_6_ (Fig. S4–S7†). To get the interlocked cage 1, L·HNO_3_ was self-assembled with a *cis*-blocked 90° Pd(ii) acceptor M in a 2 : 3 ratio at 55 °C in water for 24 hours (Scheme 1). It gave a turbid orange solution which was then centrifuged to obtain the aqueous solution of 1. Slow vapor diffusion of acetone into the aqueous solution of the product yielded pure crystals of 1.

**Fig. 1:**
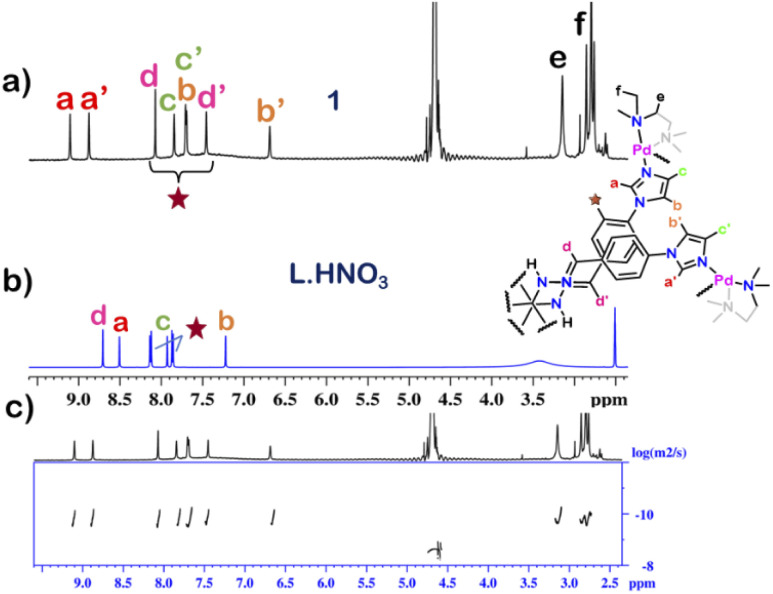
Stacked ^1^H NMR spectra of the (a) cage 1 in D_2_O, (b) ligand L·HNO_3_ in DMSO-*d*_6_ and (c) diffusion-ordered ^1^H NMR of 1 in D_2_O.

The ^1^H NMR spectrum of 1 in D_2_O at room temperature revealed the presence of seven distinct peaks in the aromatic region (Fig. 1 and S13†). Quite surprisingly, the ligand L·HNO_3_ had only six peaks (Fig. S4†) in its NMR spectrum. The presence of an extra peak in the self-assembled product suggested the formation of either an irregular structure or multiple self-assembled products. However, the ^1^H DOSY NMR spectrum showed that all the peaks correspond to same diffusion coefficient [*D* = 1.58 × 10^−10^ m^2^ s^−1^ (log *D* = −9.8)] (Fig. 1 and S20†), which confirmed the formation of a single self-assembled product.

To better understand the stoichiometry of the self-assembled product, ESI-MS spectrum of the PF_6_^−^ analogue was recorded in acetonitrile. The spectrum showed multiple peaks at *m*/*z* = 1635.957, 1190.222, 923.390 due to the charged fragments [M_6_L_4_(PF_6_)_9_]^3+^, [M_6_L_4_(PF_6_)_8_]^4+^ and [M_6_L_4_(PF_6_)_7_]^5+^ (Fig. S21 and S22†). This confirmed a [6 + 4] self-assembly of the ditopic acceptor (M) and tridentate planar donor (L). Such a combination of the donor and acceptor may yield either an interlocked structure or a double-square or an octahedral architecture.^26^

To understand the exact nature of architecture formed, ^1^H–^1^H COSY, NOESY and ^1^H–^13^C HSQC NMR of 1 were recorded in D_2_O (Fig. S16–S19†) and compared with that for L·HNO_3_ in DMSO-*d*_6_ (Fig. S5–S7†). Detailed analysis showed that the two most downfield shifted proton peaks in the ^1^H NMR spectrum of 1 at 9.10 and 8.87 ppm are that from the α-imidazole protons in L·HNO_3_ (Fig. 1). The integration of these two peaks showed that they were in a 1 : 1 ratio (Fig. S13†). If the architecture was a double-square, the α-imidazole peaks are expected to split in a 1 : 2 ratio. While if the architecture is an interlocked cage, the α-imidazole peaks are expected to split in a 1 : 1 ratio as observed.^27^

**Fig. 2:**
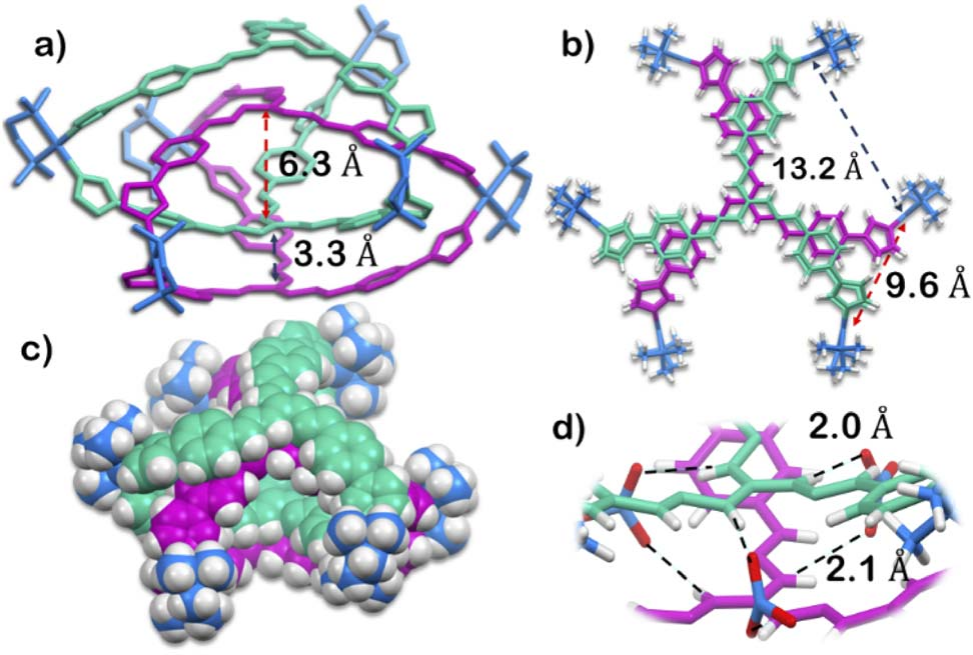
X-Ray crystal structure of 1. (a) Side view showing the inter-ligand distance of the interior cavity (H atoms and anions removed for clarity). (b) Top view showing the inter Pd distance (anions removed for clarity). (c) Side view (space fill model; anions removed for clarity). (d) Nitrate binding ability of 1 using the guanidine core. [Colour scheme: H, white; O, red; N of nitrate, blue; C, N of ligand with Δ orientation, green; C, N of ligand with Λ orientation, violet; C, N, Pd of acceptor unit, blue].

The structure was unequivocally established by single-crystal XRD (Fig. 2 and S1–S3†). Diffraction quality single crystals of 1 were grown by the slow vapour diffusion of acetone into a concentrated aqueous solution of 1 (with NO_3_^−^ as the counterion). X-ray diffraction data were collected by using a synchrotron radiation source.^28^1 crystalized in the trigonal space group *P*3̄*c*1 as a triply interlocked M_6_L_4_ cage. Unlike the known M_6_L_4_ interlocked cages,^23^1 has an asymmetrical disposition of the ligands with an easily assessable inner cavity. The crystal structure of 1 revealed that the interplanar distances between the different ligands are 3.3 Å in the periphery and 6.3 Å in the centre. The distances between two adjacent Pd centres are 9.6 Å and 13.2 Å (Fig. 2). These dimensions indicate that 1 has an internal cavity big enough to accommodate guest molecules.^27^ This was unique as interlocked structures generally do not have internal cavity big enough for guest encapsulation.^29^

**Fig. 3:**
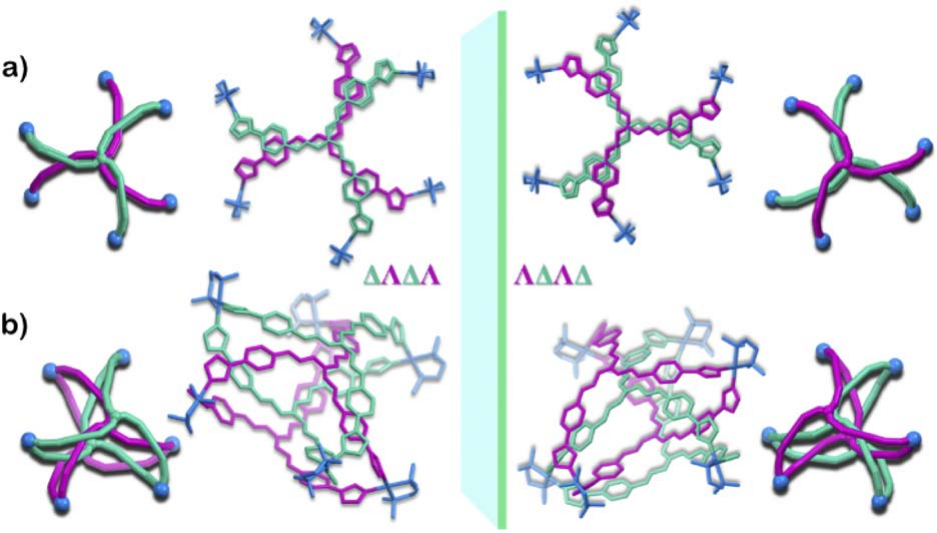
Crystal structure of 1 showing the enantiomeric relation of the two isomers ΔΛΔΛ and ΛΔΛΔ, a graphical representation is given alongside the crystal structure. (a) Top view (b) side view (H atoms and anions omitted for clarity). [Colour codes: C, N of ligand with Δ orientation, green; C, N of ligand with Λ orientation, violet; C, N, Pd of acceptor unit, blue.].

Owing to the propeller shape of the guanidine core, the interlocked cage also has a helical structure with two different orientations (Δ and Λ) (Scheme 1). The crystal structure shows that each cage is composed by the interlocking of two M_3_L_2_ cage subunits. Where one cage subunit has both ligands in the Δ orientation, and the other subunit is an isomeric cage, where both the ligands are oriented in a Λ orientation. Each subunit could thus be termed as either a ΔΔ cage or a ΛΛ cage. The M_3_L_2_ subunit (ΔΔ or ΛΛ cage) is achiral due to the presence of a plane of symmetry perpendicular to the *C*_3_ principal axis (Fig. S2†). Interestingly, due to interlocking this plane of symmetry is lost and the resultant interlocked cage 1 is chiral. Using the same notation as that of the subunits, these two enantiomers can be termed as ΔΛΔΛ or ΛΔΛΔ (Fig. 3). The crystal structure of 1 also shows that in each unit cell four molecules of 1 are present, of which two are the ΔΛΔΛ isomers and the other two are the ΛΔΛΔ isomers (Fig. S1†). This however meant that 1 is a racemic mixture containing equal amount of both the isomeric cages. The chirality of the interlocked cage 1, where the interlocking of achiral subunits resulted in the formation of a chiral interlocked structure, is noteworthy.

Finally, the crystal structure also showed the hydrogen bonding capability of the cage 1. Two N–H groups in the guanidine core of two ligand participate in H-bonding interaction with one nitrate ion. This nitrate ion has an orthogonal orientation to the two ligands. As there are three N–H groups in a ligand, and 1 comprised of four ligands, it participates in H-bonding interaction with six nitrate ions (Fig. 2 and S2†). Such H-bonding was also a unique consequence of the interlocked structure of 1.

Although cage 1 is robust and stable to other organic solvents. The cage formation reaction only occurs in water. In other solvents like DMSO or acetonitrile multiple polymeric or oligomeric species were formed. Similarly, use of acceptor (M) with other counterions such as PF_6_^−^ or triflate led to the formation of multiple species which could not be characterised by ^1^H NMR or ESI-MS. Thus, the H-bonding interaction with NO_3_^−^ coupled with the hydrophobic effect arising due to the use of aqueous medium, is the most probable driving force behind the formation of this triply interlocked cage (1) in water.

### Guest encapsulation studies

**Fig. 4:**
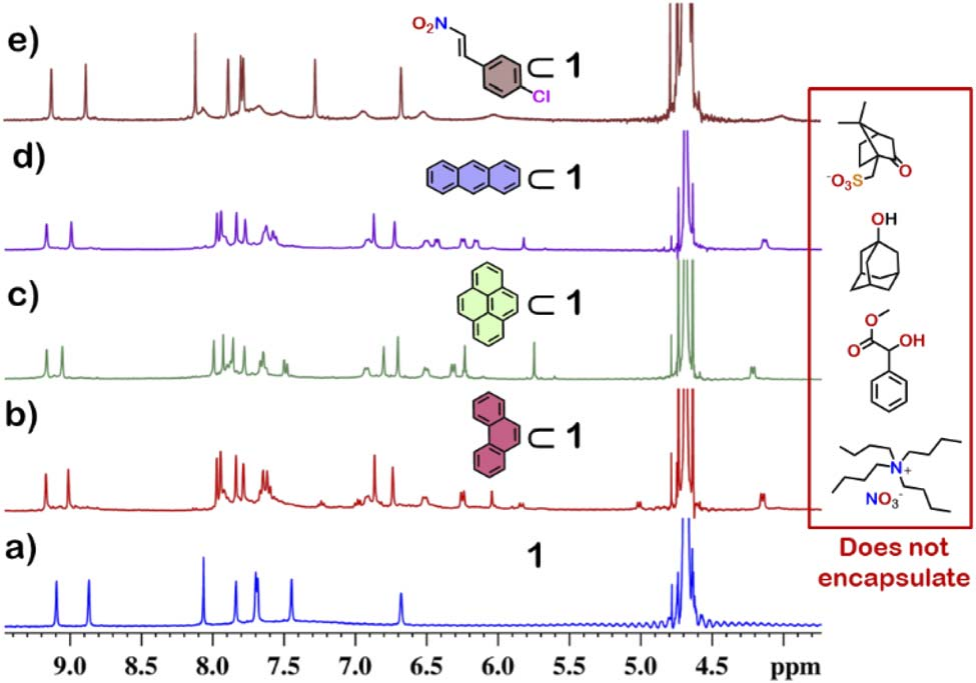
Partial ^1^H NMR stack plot of (a) 1, (b) P⊂1 (P: phenanthrene), (c) pyrene⊂1, (d) A⊂1 (A: anthracene) and (e) Ns⊂1 (Ns: (*E*)-1-chloro-4-(2-nitrovinyl)benzene) in D_2_O showing the change in NMR by guest encapsulation. (Inset) examples of non-planar guests that could not be encapsulated by 1.

From the crystal structure, 1 is expected to have two different guest binding sites. One is interior binding of guest into the inner cavity of 1. The other is exterior binding using H-bonding interactions as shown for nitrate ions. To investigate the guest binding abilities of 1, 4-chloro-β-nitrostyrene (Ns) was taken as a model guest molecule. Ns has a nitro group which could participate in H-bonding like the nitrate ion. It also has a hydrophobic styrene moiety which could favour encapsulation inside 1. To check this, excess (*ca.* 5 equivalents) of solid Ns was added to a solution of 1 in D_2_O. This mixture was stirred for 12 hours and then centrifuged to remove excess of Ns. This solution was then used to characterise the host–guest complex Ns⊂1. The ^1^H NMR of Ns⊂1 showed new peaks in 6–7 ppm region (Fig. 4 and S23†), with such a shift of the guest peaks characteristic of interior binding. Further proof of binding was obtained from the ^1^H DOSY spectrum of Ns⊂1 in D_2_O. It showed that all peaks correspond to a single diffusion band (log *D* = −10) (Fig. S24†). However, because of the broad nature of the guest peaks, host–guest stoichiometry could not be accurately determined. We thus investigated the host–guest behaviour with other planar guests.

It was found through ^1^H NMR analysis that 1 was able to encapsulate many polycyclic aromatic hydrocarbons (PAHs) like pyrene, phenanthrene (P) and anthracene (A) (Fig. 4 and S25†). Much like with Ns, it was seen that for both P⊂1 and A⊂1, encapsulation took place in the inner cavity of 1. All the ^1^H NMR peaks for P⊂1 had a single diffusion coefficient (log *D* = −9.8) (Fig. S26†) and ^1^H NOESY spectrum showed correlation between host and guest peaks (Fig. S27†), both characteristic for interior guest encapsulation. Similar results were also obtained for A⊂1 (Fig. S28 and S29†). However, as 1 is chiral encapsulation of the guest inside 1 led to loss of symmetry of the guest and several new peaks for the host–guest complex are observed. To accurately determine the host–guest ratio, titration experiments were carried out for A⊂1 and P⊂1.

To perform the ^1^H NMR titration a stock solution of 1 was prepared in 0.5 mL D_2_O with tetrabutylammonium nitrate [(N(Bu)_4_)NO_3_] as the internal standard. As both P and A are insoluble in water, stock solutions of P in CD_3_OD (0.017 M) and A in DMSO-*d*_6_ (0.017 M) were prepared. The ^1^H NMR titration was performed by adding aliquots of guest stock solution (10 μL) to the stock solution of 1. After each addition ^1^H NMR was recorded without any time delay. It was found that both the guests (A and P) were bound to 1 by slow exchange on the NMR time scale at 25 °C (Fig. S30 and S32†). Thus, the host–guest concentration [HG] could be determined by integration with respect to the internal standard. The plot of [HG] *vs.* equivalence of guest added showed that saturation in concentration of [HG] was achieved when *ca.* 1 equivalent of either A or P was added (Fig. S31 and S33†). From this we could roughly conclude that for both the systems A⊂1 and P⊂1, the host to guest stoichiometry is 1 : 1. From the ^1^H NMR titration, the apparent association constant could also be calculated using the Hill equation.^30^ The apparent association constants for the formation of A⊂1 and P⊂1 were found to be 9.03 × 10^2^ M^−1^ (Fig. S31†) and 6.42 × 10^3^ M^−1^ (Fig. S33†), respectively.^31^

**Fig. 5:**
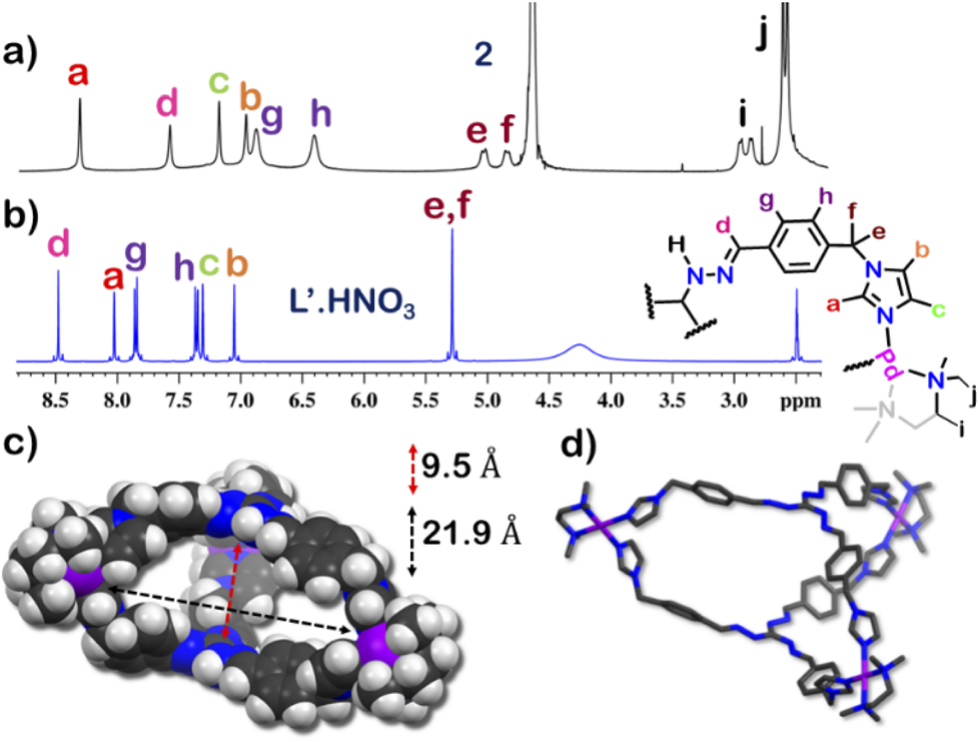
Stacked ^1^H NMR spectra of (a) cage 2 in D_2_O, (b) ligand L′.HNO_3_ in DMSO-*d*_6_ and (c) optimized structure of 2 (PM6 level), side view (space fill model) showing the inter ligand distance and inter Pd distance of the interior cavity. (d) Side view (capped-stick model) (H-atoms were removed for clarity) [colour scheme: H, white; C, black; N, blue; Pd, violet].

The guest binding ability of 1 was unique and thus we wanted to check the importance of the interlocked structure towards this property. To check this we constructed a non-interlocked cage 2 using ligand L′.HNO_3_ with the same guanidine core (Fig. 5). Unlike L·HNO_3_ which had rigid imidazole donors, L′·HNO_3_ has flexible imidazole arms (Fig. S9–S11†). Self-assembly of L′·HNO_3_ with acceptor M in a 2 : 3 ratio in water gave an orange solution. Complex 2 was first characterized by ^1^H NMR (Fig. 5 and S34†), which showed the presence of six peaks in the aromatic region with an NMR pattern similar to that for L′·HNO_3_ in DMSO-*d*_6_. Absence of extra peak in the ^1^H NMR spectra indicated the formation of a non-interlocked structure. All the peaks of 2 correspond to the same diffusion coefficient (log *D* = −9.3) in the DOSY NMR which confirmed the formation of a single assembly.

To better understand the stoichiometry of the self-assembled product, ESI-MS spectrum of the PF_6_^−^ analogue was recorded in acetonitrile. The spectrum showed multiple peaks at *m*/*z* = 1232.343, 773.240, 543.686 correspond to the charged fragments 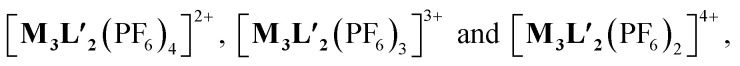 respectively (Fig. S38 and S39†). This confirmed that introduction of flexibility in the ligand backbone led to the formation of a 
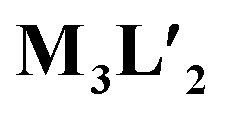
 architecture. Complex 2 formed very tiny crystals upon slow vapor diffusion of acetone into a concentrated aqueous solution of the cage. These could not be used for X-ray structure elucidation, thus the structure of 2 was optimized semi-empirically using the PM6 model. The optimized structure showed that 2 has a large inner cavity with an inter ligand distance of 9.5 Å and a 21.9 Å distance between two Pd atoms (Fig. 5).

If the encapsulation behavior exhibited by 1 was a result of its ligand backbone, 2 is also expected to show same host–guest property. This however was not the case as 2 was found to show no affinity towards any guests (planar or non-planar). This proved that the interlock structure of 1 was pivotal for guest encapsulation. The inability of 2 to encapsulate any guest is probably because of its big pocket size which did not facilitate interactions strong enough to stabilize a guest inside its cavity.

### Selective guest encapsulation: planar *vs.* non-planar

**Fig. 6:**
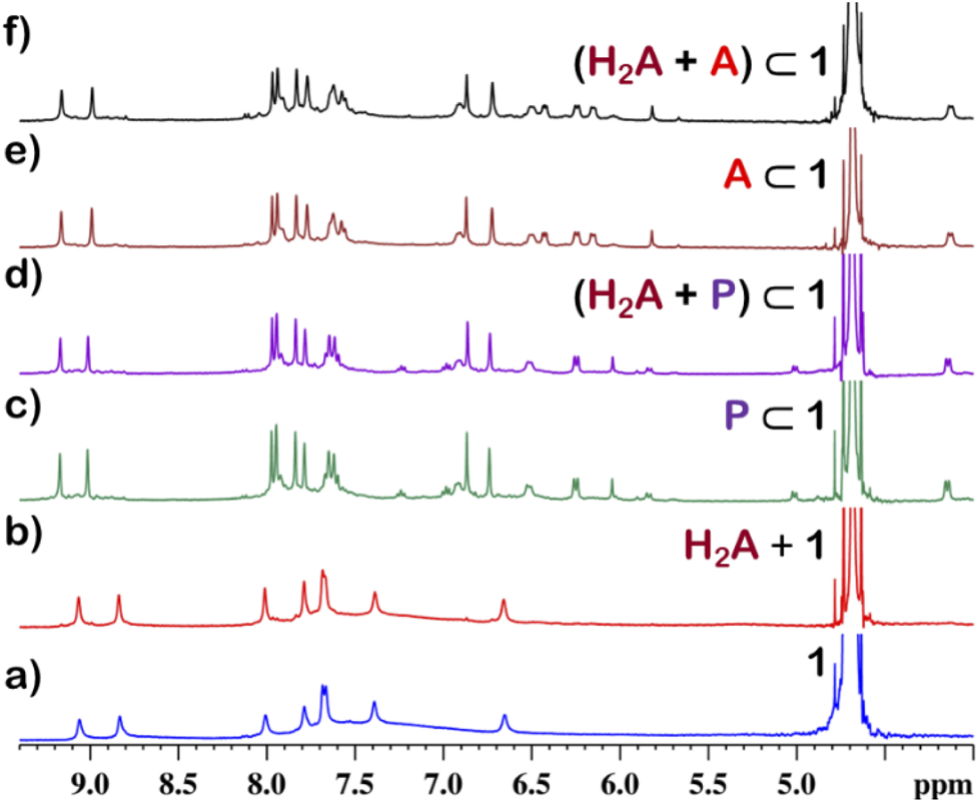
Partial ^1^H NMR stack plot of (a) 1, (b) (H_2_A + 1) (H_2_A = 9,10-dihydroanthracene), (c) P⊂1 (P: phenanthrene), (d) (H_2_A + P)⊂1, (e) A⊂1 (A: anthracene) and (f) (H_2_A + A)⊂1 in D_2_O showing the selective encapsulation of A and P.

Although 1 could encapsulate planar hydrocarbons like anthracene and phenanthrene, it did not encapsulate molecules with adamantane or camphor moiety (Fig. 4). Such observation was very interesting and led to the idea that 1 could be used for the separation of planar and non-planar hydrocarbons. Such separation is highly challenging in coal industry, especially the separation of 9,10-dihydroanthracene (H_2_A) from its planar analogues anthracene (A) and its isomer phenanthrene (P). Further, small scale (milligram level) separation is even more difficult due to almost same *R*_f_ values of these compounds. To check this, first encapsulation of H_2_A by 1 was studied. Excess of solid H_2_A (*ca.* 5 equivalents) was added to a D_2_O solution of 1 and stirred for 12 hours. Then the solution was centrifuged to remove excess guests and the ^1^H NMR of the supernatant was recorded. The ^1^H NMR of H_2_A + 1 was found to be identical to that of 1 (Fig. 6). This proved that 1 did not encapsulate H_2_A.

**Scheme 2:**
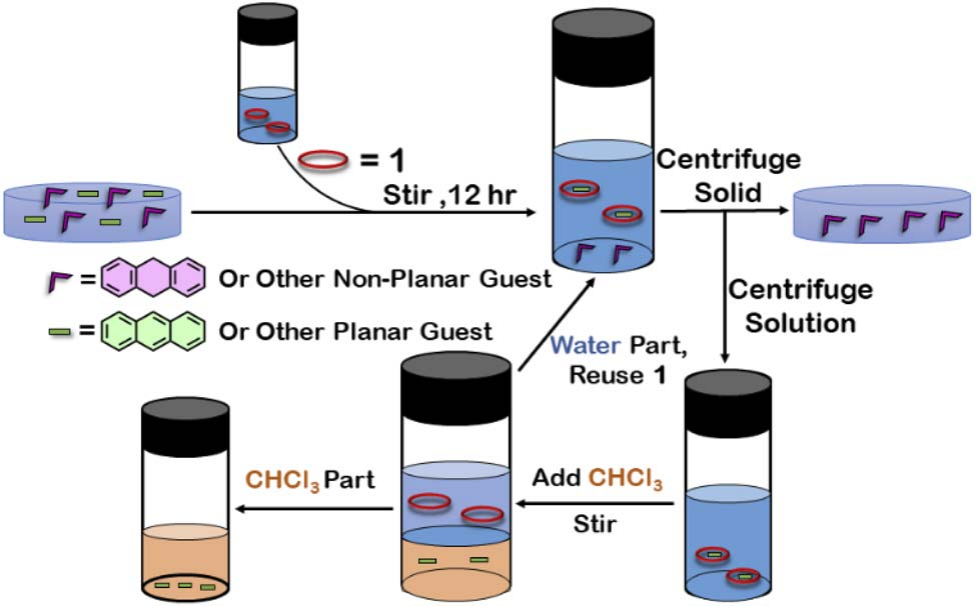
Schematic representation for the separation of planar and non-planar hydrocarbons achieved by employing host–guest interactions with 1.

To examine the selective guest uptake, a solid mixture of equimolar H_2_A and A was added to a D_2_O solution of 1. The resultant solution was stirred for 12 hours and then centrifuged to remove excess guests (Scheme 2). The ^1^H NMR of the supernatant was identical to that of A⊂1 (Fig. 6 and S40†). This proved that from the mixture only anthracene was encapsulated selectively. Similar selective encapsulation of planar phenanthrene (P) was noticed when equimolar mixture of H_2_A and P was added to 1 (Fig. S41†). It also showed selective encapsulation of phenanthrene over 9,10-dihydroanthracene. To further verify this, the guest was extracted using chloroform. The extracted organic solvent was removed, and the resultant white solid was redissolved in 0.5 mL CDCl_3_. The ^1^H NMR of the extracted solid gave the peaks corresponding to only phenanthrene. This further verified the claim that in the host–guest complex only phenanthrene was present as guest (Fig. S42†).

To check if 1 has a general tendency to separate planar and non-planar molecules. Selective encapsulation was carried out in the presence of two other non-planar guests, thianthrene (S_2_A) and *N*-methylphenothiazine (MP). Following the same experiments as employed for H_2_A, it was seen that both S_2_A and MP were not encapsulated by 1. Further selective encapsulation experiments showed that from an equimolar mixture of S_2_A and A, selectively only A was encapsulated (Fig. S43†). Similar results were also obtained for an equimolar mixture of MP and A (Fig. S44†). Titration of 1 with H_2_A, S_2_A and MP also showed no change in ^1^H NMR (Fig. S45–S47†) which further demonstrated the fact that 1 did not encapsulate these non-planar molecules. This clearly demonstrated that 1 successfully separated planar anthracene from its non-planar derivatives.

## Conclusions

In conclusion, we have successfully synthesized a water-soluble interlocked cage (1) by metal–ligand coordination self-assembly of propeller shaped tridentate ligand L·HNO_3_ with *cis*-blocked Pd(ii) 90° ditopic acceptor M. 1 has a triply interlocked structure with an internal cavity capable of guest uptake and release without damaging the integrity of the cage. This internal cavity was found to encapsulate planar aromatic guests like anthracene and phenanthrene. The inclusion complexes were characterized by several multinuclear NMR studies, and the host–guest ratio for the inclusion complexes was found to be 1 : 1. Further investigations revealed that this guest encapsulation ability was resulted from the interlocked structure, whereas a non-interlocked cage 2 with similar backbone did not show any guest encapsulation. Separation of planar polycyclic aromatic hydrocarbons from their non-planar hydrogenated analogues is a challenging problem in petrochemical industries, especially the separation of non-planar 9,10-dihydroanthracene from planar phenanthrene or anthracene by fractional distillation at high temperature. Surprisingly, the interlocked cage (1) was successful in the separation of such systems in water by selective encapsulation of planar molecules followed by their de-encapsulation using organic solvent at ambient condition. Such guest encapsulation and de-encapsulation by an interlocked cage 1 is an unusual observation as interlocked systems are either reluctant towards guests due to a lack of internal cavity or degrade if the guest is removed in the cases where the interlocked systems are formed in the presence of a guest as template. Our present investigations show a simple strategy for separating planar and non-planar hydrocarbons from their solid mixture by aqueous extraction using an interlocked cage as extracting agent at ambient condition. It also offers a new application of water-soluble interlocked cage apart from their aesthetic beauty.

## Data availability

All data are provided in the ESI† and additional data can be available upon request.

## Author contributions

P. S. M and D. C. devised the project and designed the experiments. D. C. carried out the the experimental work together with R. S., and analyzed the data. J. K. C. collected and solved the crystallographic data. P. S. M. supervised the whole project. All authors contributed to the writing of the manuscript.

## Conflicts of interest

There are no conflicts to declare.

## Supplementary Material

SC-013-D2SC04660A-s001

SC-013-D2SC04660A-s002
