# Supplementary material for: Selective separation of planar and non-planar hydrocarbons using an aqueous Pd6 interlocked cage
Source: Chem Sci. 2022 Sep 14;13(40):11764–71. doi: 10.1039/d2sc04660a (PMC9580621; doi:10.1039/d2sc04660a)
Supplement: SC-013-D2SC04660A-s001 [file SC-013-D2SC04660A-s001.pdf]

Electronic Supplementary Material (ESI) for Chemical Science.
This journal is © The Royal Society of Chemistry 2022

Supporting Information

Selective separation of planar and non-planar hydrocarbons using an aqueous Pd_6 interlocked cage

Debsena Chakraborty,[a] Rupak Saha,[a] Jack K. Clegg[b] and Partha Sarathi Mukherjee[a]*

a Department of Inorganic and Physical Chemistry
Indian Institute of Science,
Bangalore 560012 (India).
E-mail: psm@iisc.ac.in

b School of Chemistry and Molecular Biosciences
The University of Queensland,
St. Lucia, Queensland 4072 (Australia).

Contents:

1	Materials and methods	2
2	X-Ray crystallographic study of **1**	4
3	Spectral characterization of **L.HNO$_3$** & **L'.HNO$_3$**	7
4	Spectral characterization of **1**	10
5	Host-Guest chemistry of **1**	13
6	Spectral characterization of **2**	19
7	Selective host-guest chemistry	21
8	Optimized structures	26
9	References	52

1.1 Materials and methods:

General chemicals and the solvents were purchased from commercially available suppliers and were used without further purification. All the reactions were carried out under ambient conditions in normal atmosphere. The NMR spectra of the newly prepared materials were recorded on BRUKER 400 MHz and 500 MHz spectrometers. The chemical shifts (δ) in the ^{1}H NMR spectra were reported in ppm relative to the tetramethylsilane, which was used as an internal standard (δ = 0.00 ppm) or the resonance of the proton resulting from partial deuteration of the NMR solvents: D2O (δ = 4.79 ppm), $CDCl_3$ (δ = 7.26 ppm), CD_3CN (δ = 1.94 ppm) and DMSO-d_6 (δ = 2.50 ppm). ^{13}C NMR spectra were recorded using the same instruments at 100 MHz, 125 MHz and all the chemical shifts (δ) were reported in ppm relative to external $CDCl_3$ at 77.8-77.2 ppm, CD_3CN at 1.32, 118.26 ppm and DMSO-d_6 at 39.52 ppm. Electrospray ionization mass spectra were recorded using Agilent 6538 Ultra-High Definition (UHD) Accurate Mass Q-TOF spectrometer along with the use of standard spectroscopic grade solvents. Electronic absorption spectra were recorded on a LAMBDA 750 UV/Vis spectrophotometer.

1.2 Synthetic routes:

1.2.1 Synthesis of ligand L·HNO_3

A solution of 4-(1H-Imidazol-1-yl)benzaldehyde (1.08 g, 6.28 mmol) and ethanol (30 mL) was added dropwise to a 10 mL aqueous solution of triaminoguanidinium nitrate (300.0 mg, 5 mmol). The afforded solution was allowed to reflux for 24 hours which resulted in the formation of a yellow precipitate after around 4 hours. The precipitate was then filtered and washed with ice-cold ethanol (2 times) and then with 20 mL of acetone. Finally, the precipitate was kept for drying under vacuum to get the triimidazole ligand **L**·HNO_3 as a yellow powder. Isolated yield: 1 g (88%). ^{1}H NMR (500 MHz, DMSO-d_6): δ (ppm) 8.71 (s, 3H), 8.51 (s, 3H), 8.14 (d, 6H), 7.93 (s, 3H),7.87 (d, 6H), 7.22 (s, 3H).^{13}C NMR (125 MHz, DMSO-d_6): δ (ppm) 150.01, 149.73, 138.85, 136.01, 132.23, 130.21, 129.99, 120.82, 118.50. HRMS (ESI): $C_{31}H_{26}N_{12}(HNO_3)$, $[M+H]^+$ = 567.247 (calculated) Found: 567.246 .

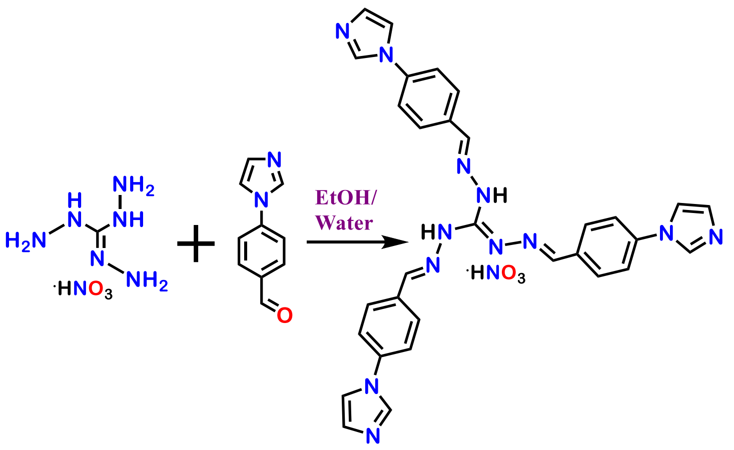

Scheme S1: Synthetic route for the preparation of the ligand **L.HNO$_3$**.

1.2.2 Synthesis of ligand L'·HNO_3

The ligand **L'**·HNO_3 was prepared using a similar process as employed for **L**·HNO_3. Only, instead of 4-(1H-Imidazol-1-yl)benzaldehyde, 4-((1H-imidazol-1-yl)methyl)benzaldehyde[1] (1.00 g, 5.37 mmol) was used and accordingly 256.0 mg (1.53 mmol) of triaminoguanidinium nitrate was used. Isolated yield: 850 mg (83%). ^{1}H NMR (400 MHz, DMSO-d_6): δ (ppm) 8.48 (s, 3H), 8.02 (s, 3H), 7.86 (d, 6H), 7.37 (d, 6H), 7.31 (s, 3H), 7.05 (s, 3H), 5.29 (s, 6H).^{13}C NMR (100 MHz, DMSO-d_6): δ (ppm) 151.54, 148.18, 139.96, 138.07, 134.85, 128.82, 128.62, 128.30, 120.90, 50.50. HRMS (ESI): $C_{34}H_{33}N_{12}(HNO_3)$, $[M+H]^+$= 609.295 (calculated) Found: 609.297.

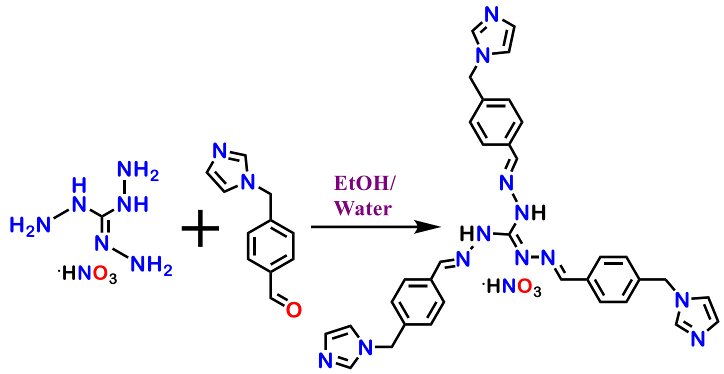

Scheme S2: Synthetic route for the preparation of the ligand **L'·HNO_3**.

1.2.3 Synthesis of interlocked cage 1

In a 4 mL glass vial, **L·HNO_3** (24.2 mg, 38.44 µmol) was added to a 2 mL Millipore water solution of *cis*-[(tmeda)Pd(NO_3)$_2$] **M** (20.0 mg, 57.64 µmol). The mixture was heated at 55 °C for 24 hours to result a turbid orange solution. The resulting mixture was centrifuged, and the clear supernatant was taken. It was further purified via crystallization by slow vapor diffusion of acetone into the aqueous solution. Yield: 7.5 µmol (78%). ^{1}H NMR (D_2O, 500 MHz): δ (ppm) 9.27(s, 6H), 9.04(s, 6H), 8.22(bs, 12H), 7.97(bs, 18H), 7.84(bs, 30H), 7.62(bs, 18H), 6.81(s, 6H), 3.27(bs, 24H), 2.98-2.89(m, 72H). ESI-MS (CH_3CN) m/z = 923.3901 for $[M_6L_4(PF_6)_7]^{5+}$ (calc. 923.3865), 1190.4746 for $[M_6L_4(PF_6)_8]^{4+}$ (calc. 1190.4742), 1635.6194 for $[M_6L_4(PF_6)_9]^{3+}$ (calc. 1635.6203).

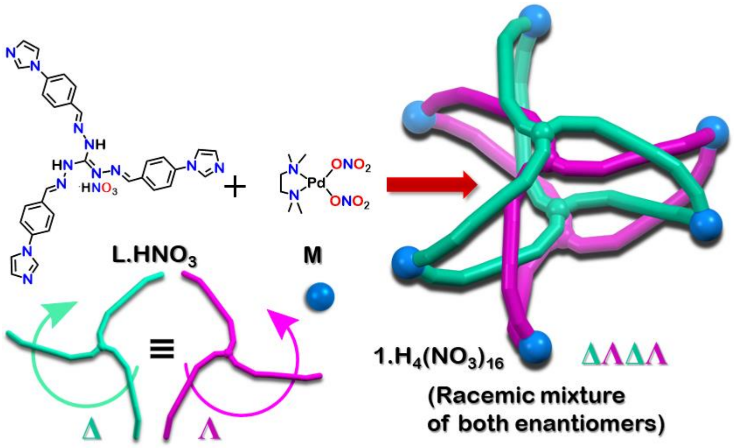

Scheme S3: Schematic representation of the synthesis of **1**.

1.2.4 Synthesis of free cage 2

In a 4 mL glass vial, **L'·HNO$_3$** (26.0 mg, 38.72 μmol) was added to a 2 mL Millipore water solution of **M** (20.1 mg, 58.04 μmol). The mixture was heated at 60 °C for 24 hours to result a clear orange solution. The solvent was then removed completely, and the resultant solid was washed with acetone. The solid was dried under vacuum to obtain **2** with relatively high purity. Yield: 40 mg (91%). ^{1}H NMR (D$_2$O, 500 MHz): δ (ppm) 8.45(s, 6H), 7.73(s, 6H), 7.33(s, 6H), 7.04(bs, 18H), 6.56(bs, 12H), 5.21(d, 6H), 5.02(d, 6H), 3.13-3.11(dd, 12H), 2.78-2.74(d, 36H). ESI-MS (CH$_3$CN) m/z = 543.6891 for $[M_3L'_2(PF_6)_2]^{4+}$ (calc. 543.6535), 773.2401 for $[M_3L'_2(PF_6)_3]^{3+}$ (calc. 773.1927), 1232.3431 for $[M_3L'_2(PF_6)_4]^{2+}$ (calc. 1232.2711).

2. X-Ray crystallographic study of 1

Single crystal X-ray data were collected using Silicon Double Crystal monochromated synchrotron radiation at 100(2) K at the MX2 beamline of the Australian synchrotron.[2] Data integration and reduction was performed using XDS.[3] The structure was solved by intrinsic phasing using ShelXT[4] and refined by the full-matrix least-squares method using SHELXL through the Olex2 GUI.[5] In general, non-hydrogen atoms with occupancies of greater than 0.5 were refined anisotropically, carbon-bound hydrogen atoms were included in idealized positions and refined using a riding model.[6] The structure has a large volume of smeared electron density in the lattice corresponding to highly disordered solvents and anions which could not be successfully modelled. This region of electron density was therefore treated with the solvent masking[6b] algorithm of Olex2. Crystallographic data and refinement parameter are given in Table S4. The CIF has been deposited with the CCDC number 2174542.

	**1**
Empirical formula	$C_{160}H_{204}N_{84}O_{72}Pd_6$
Formula weight	5094.46
Temperature (K)	100(2)
Crystal system	trigonal
Space group	P-3c1
a (Å)	27.653(4)
b (Å)	27.653(4)
c (Å)	44.488(9)
α (°)	90
β (°)	90
γ (°)	120
V (Å^3)	29463(10)
Z	4
ρcalcd (g/cm^3)	1.149
μ (mm^{-1})	0.436
F(000)	10416.0
Crystal size (mm^3)	0.100 × 0.100 × 0.100

θ (°)	2.250 – 28.284
Limiting indices	$-36 \le h \le 36$<br>$-36 \le k \le 36$<br>$-59 \le l \le 59$
No. of reflections collected	456097
No. of independent reflections (R_{int})	14626
Completeness to θ /%	99.8
No. of data/restraints/params	24357 /0/ 795
Goodness of fit (GOF) on F^2	1.066
Final *R* indices ($I > 2\,\theta\,(I)$)<br>R_1<br>$wR2$	 0.0910 [a]<br>0.2931 [b]
R indices (all data)<br>$R1$<br>$wR2$	 0.1120 [a]<br>0.3221 [b]
Largest difference in peak, hole (e Å−3)	2.202, -0.614
CCDC No.	2174542

[a] $R_1 = \Sigma(|F_o| - |F_c|)/\Sigma|F_o|$. [b] $wR_2 = \{\Sigma[w(|F_o|^2 - |F_c|^2)^2]/\Sigma[w(|F_o|^2)^2]\}^{1/2}$

Table S1: Crystallographic data and refinement parameters of **1.**

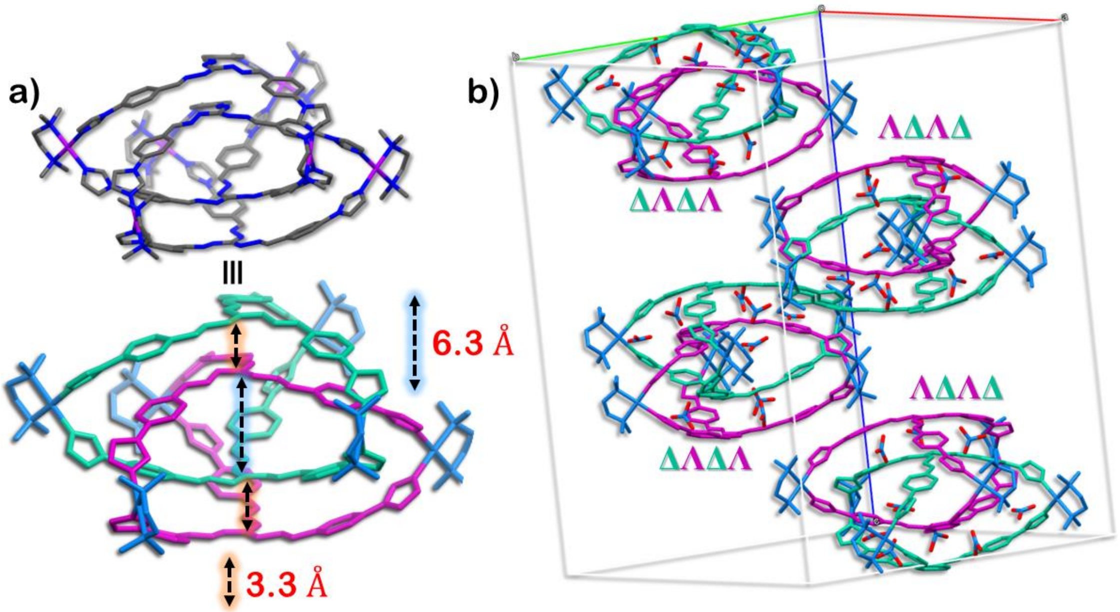

Fig. S1: Crystal structure of **1:** a) side view of one enantiomer with interplane distances b) crystal packing of 1, showing presence of both isomers in one unit cell. [Black, Blue, Violet colours denote C, N and Pd atoms (H atoms and anions omitted for clarity)].

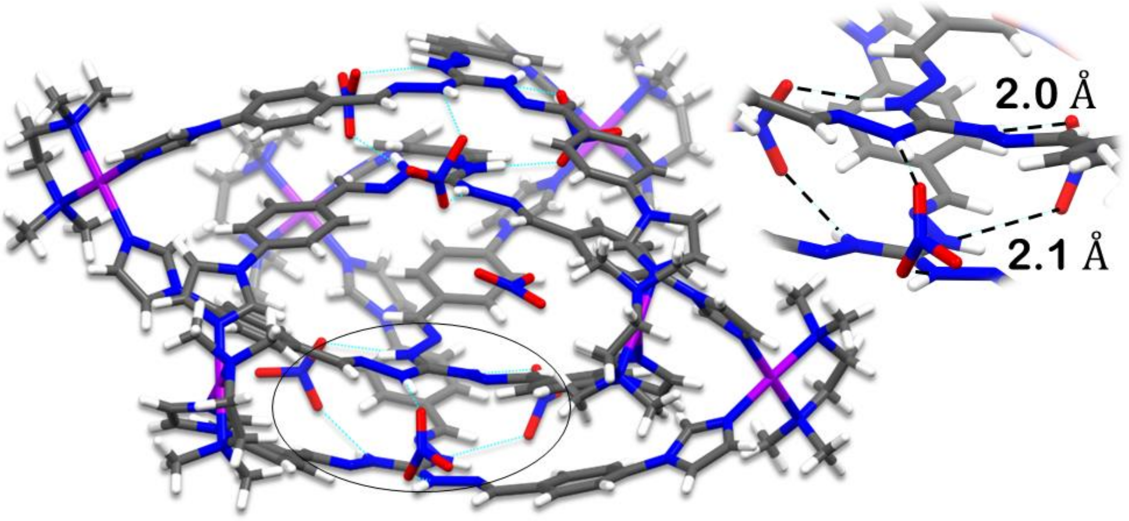

Fig. S2: Crystal structure of **1** showing the six nitrate anions bound orthogonally through H-bonding. (Inset) Magnified portion showing the H-bonding interactions involved. [Black, Blue, Red, Violet colours denote C, N, O and Pd atoms].

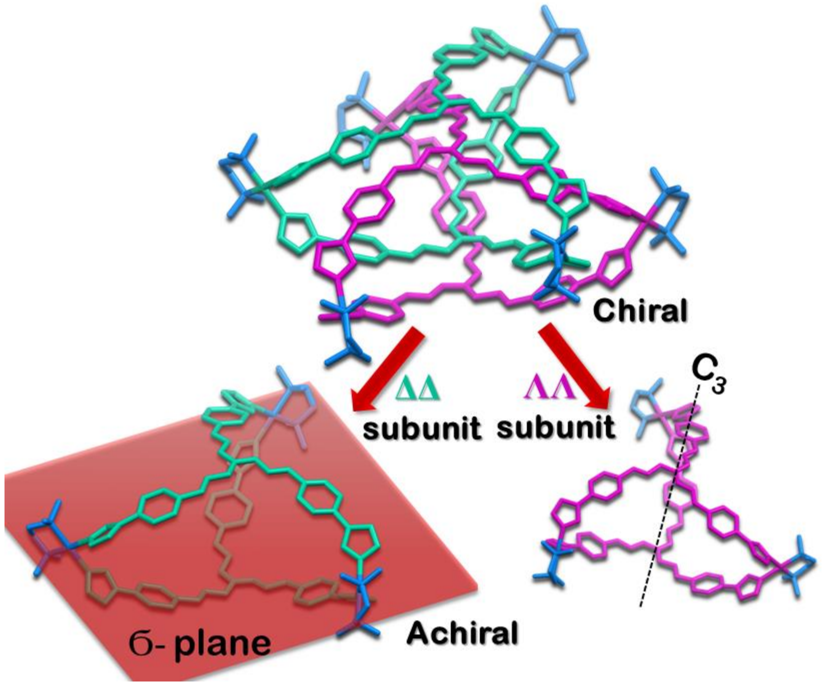

Fig. S3: Sub-components of **1** and the symmetry operations of the subunits.

3. Spectral characterization of L·HNO3 & L'·HNO3:

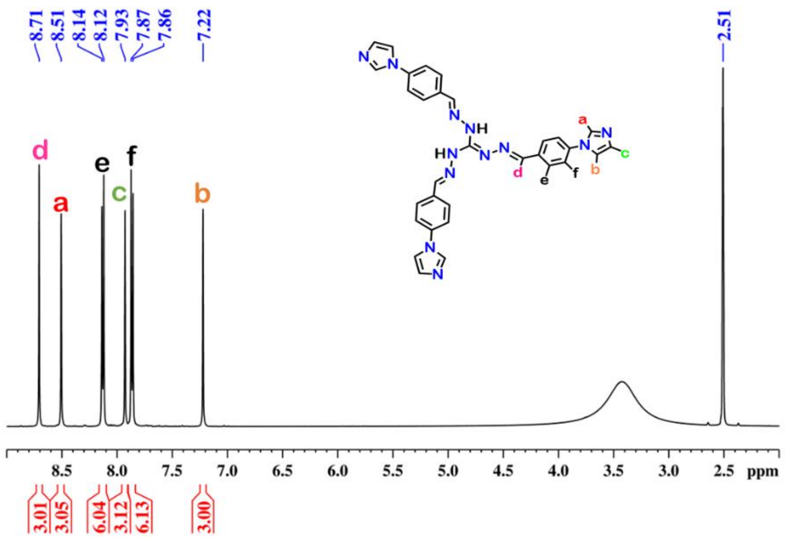

Fig. S4: ^{1}H-NMR spectrum of ligand **L·HNO3** in DMSO-*d6*.

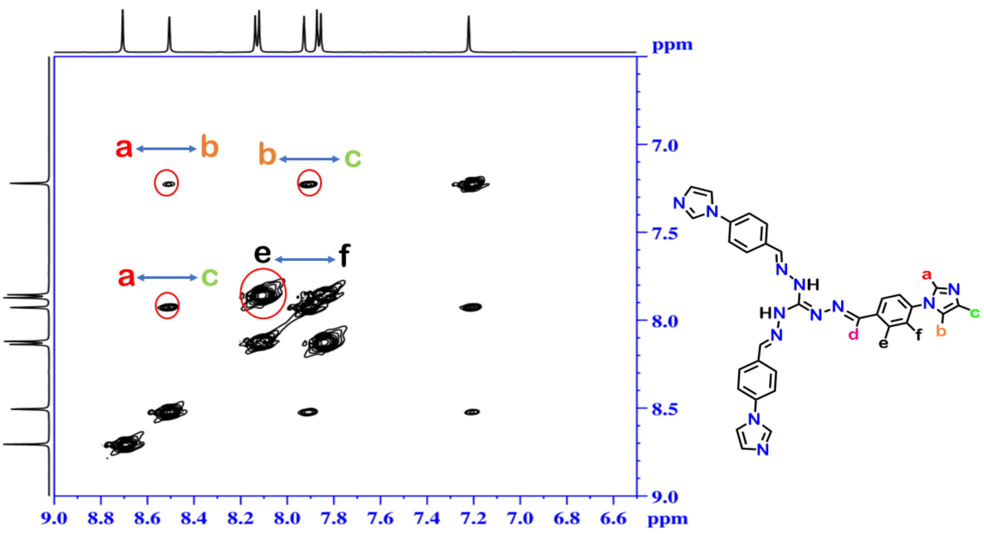

Fig. S5: Partial ^{1}H-^{1}H COSY NMR spectrum of **L·HNO3** in DMSO-*d6*.

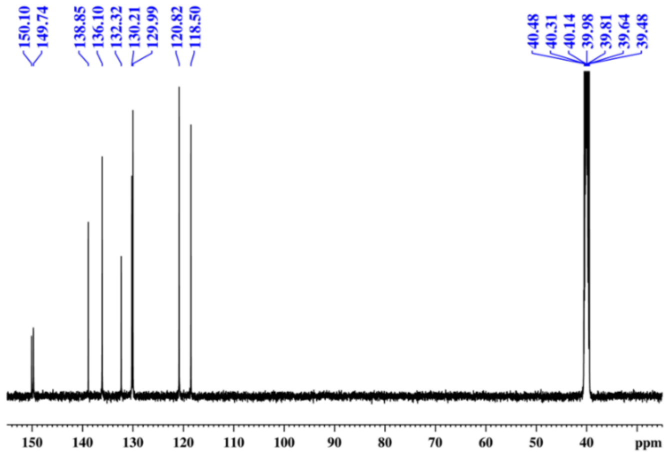

Fig. S6: ^{13}C NMR spectrum of **L·HNO3** in DMSO-*d6*.

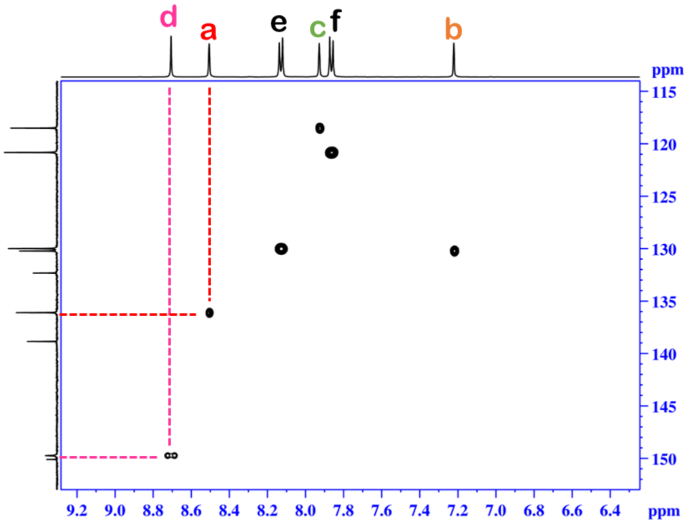

Fig. S7: ^{13}C-^{1}H HSQC spectrum of **L·HNO3** in DMSO-*d6*.

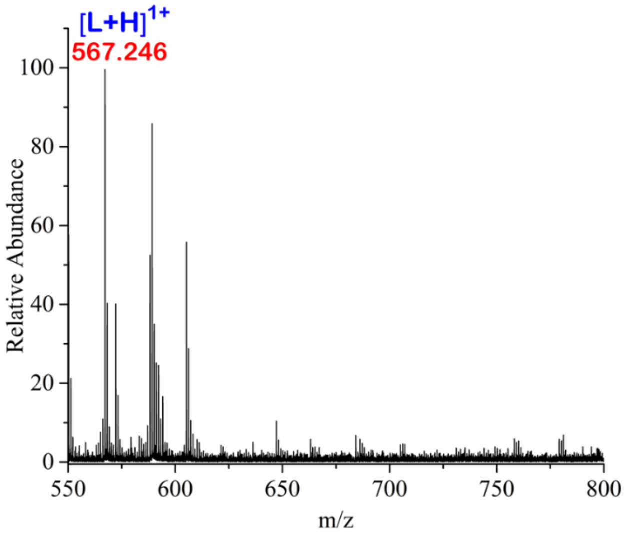

Fig. S8: Mass-spectrum of the ligand **L** in CH_3OH.

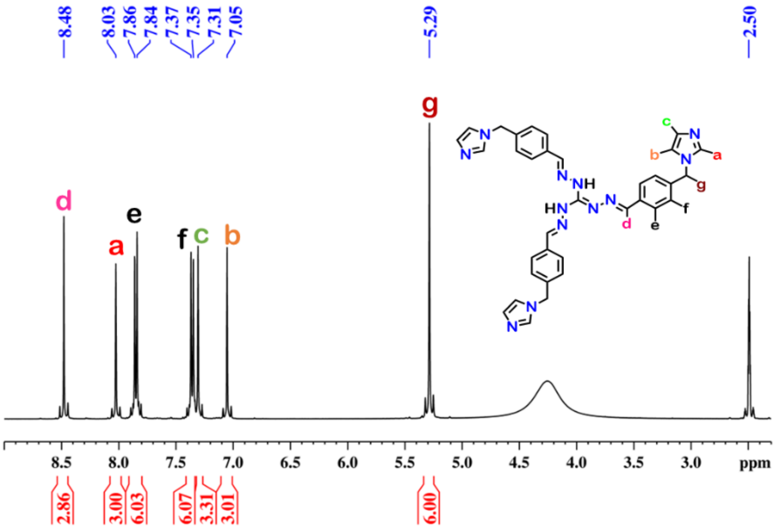

Fig. S9: ^{1}H-NMR spectrum of ligand **L'·HNO3** in DMSO-*d6*.

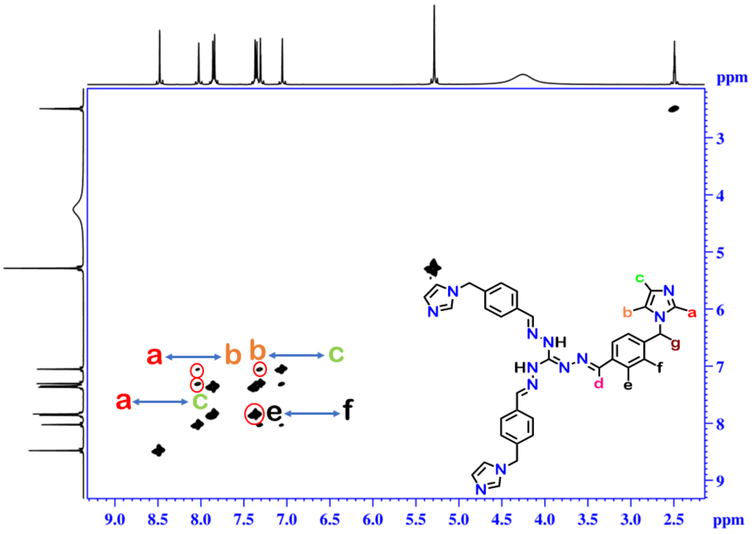

Fig. S10: ^{1}H-^{1}H COSY NMR spectrum of **L’·HNO₃** in DMSO-*d₆*.

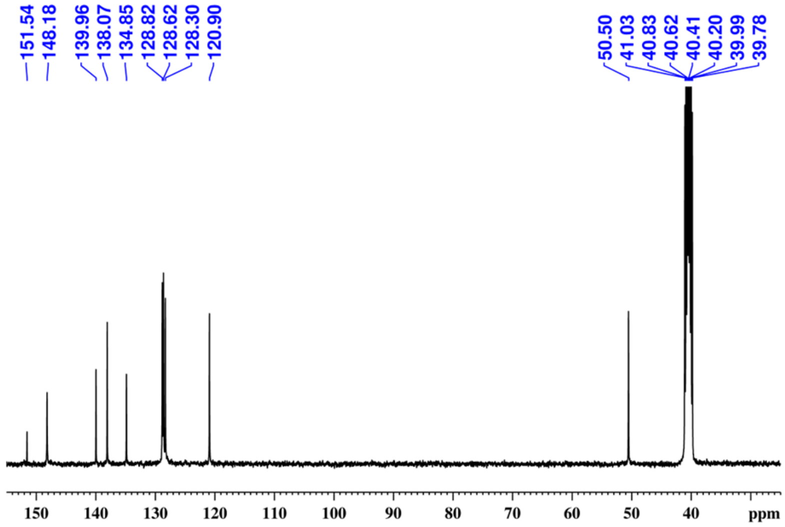

Fig. S11: ^{13}C NMR spectrum of **L’·HNO₃** in DMSO-*d₆*.

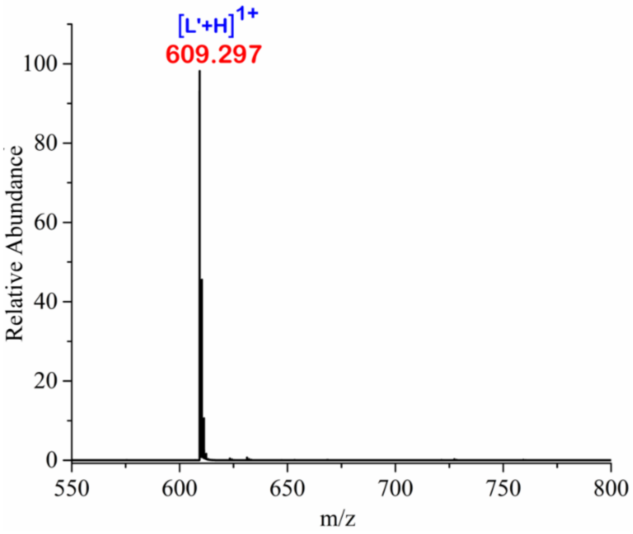

Fig. S12: Mass spectrum of the ligand **L’** in CH_3OH.

4. Spectral characterization of 1:

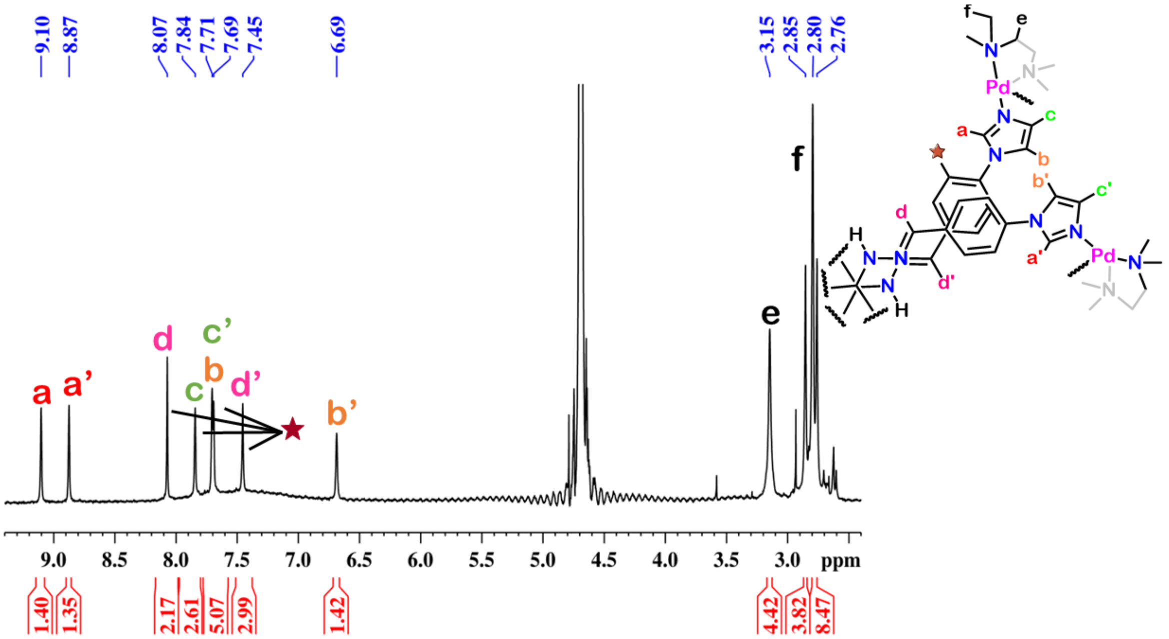

Fig. S13: ^{1}H NMR spectrum of **1** in D_2O.

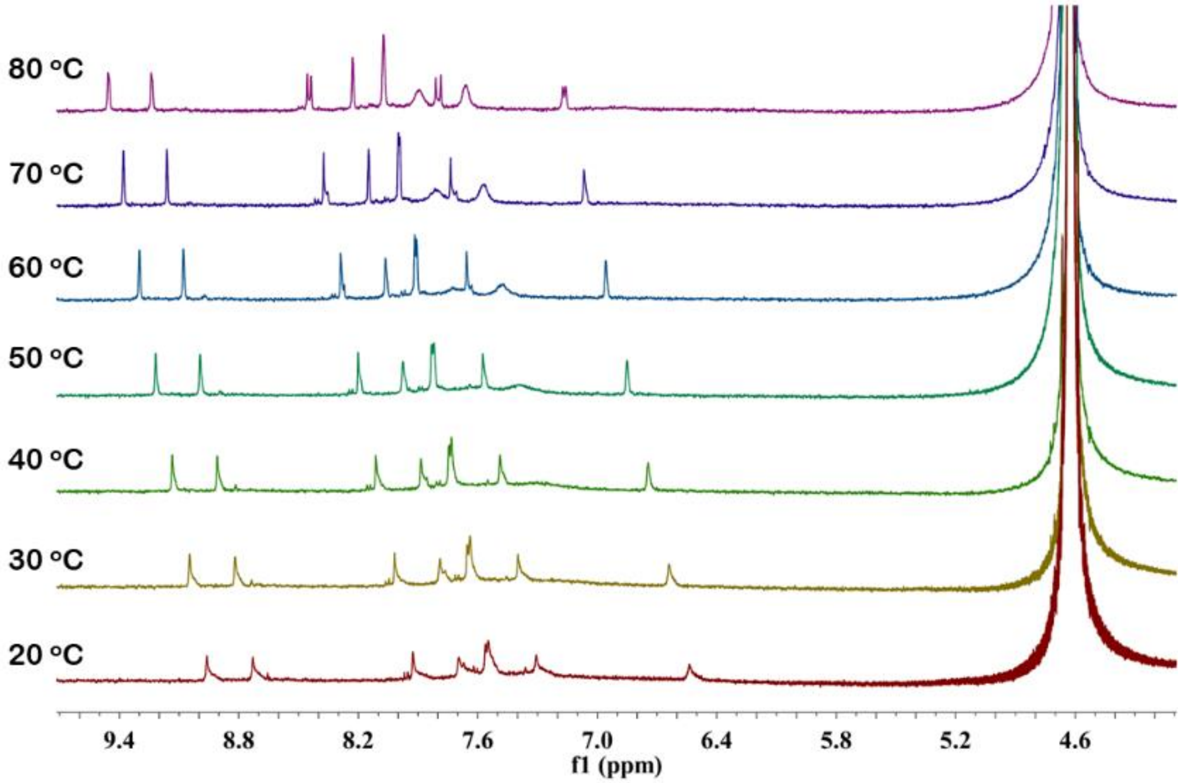

Fig. S14: ^{1}H Variable temperature ^{1}H NMR spectra of **1** in D_2O showing the appearance of phenyl peaks at higher temperature.

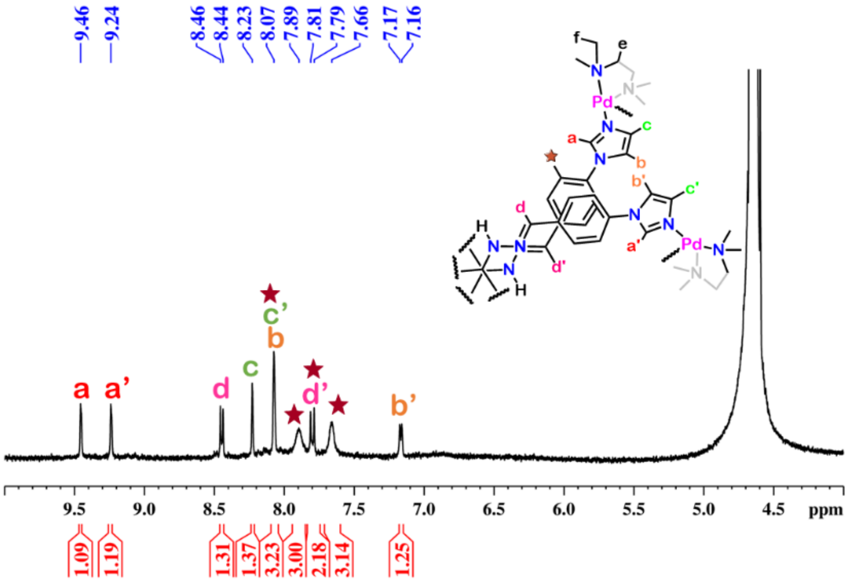

Fig. S15: ^{1}H NMR spectrum of **1** in D_2O at 80°C.

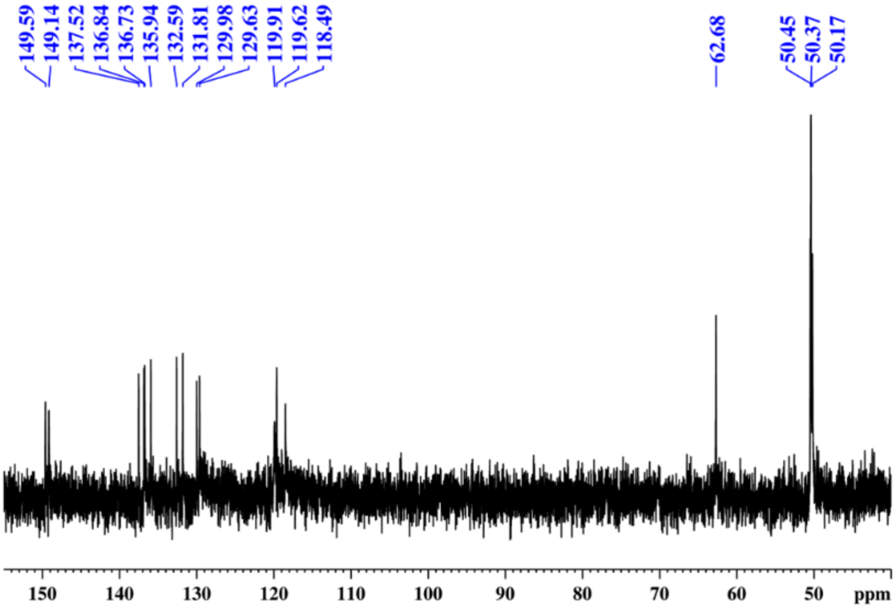

Fig. S16: ^{13}C NMR spectrum of **1** in D_2O.

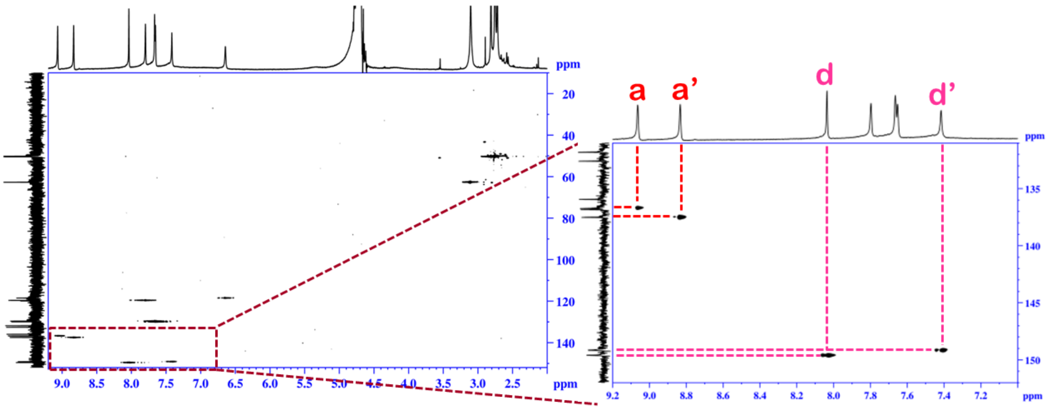

Fig. S17: ^{13}C-^{1}H HSQC spectrum of **1** in D_2O.

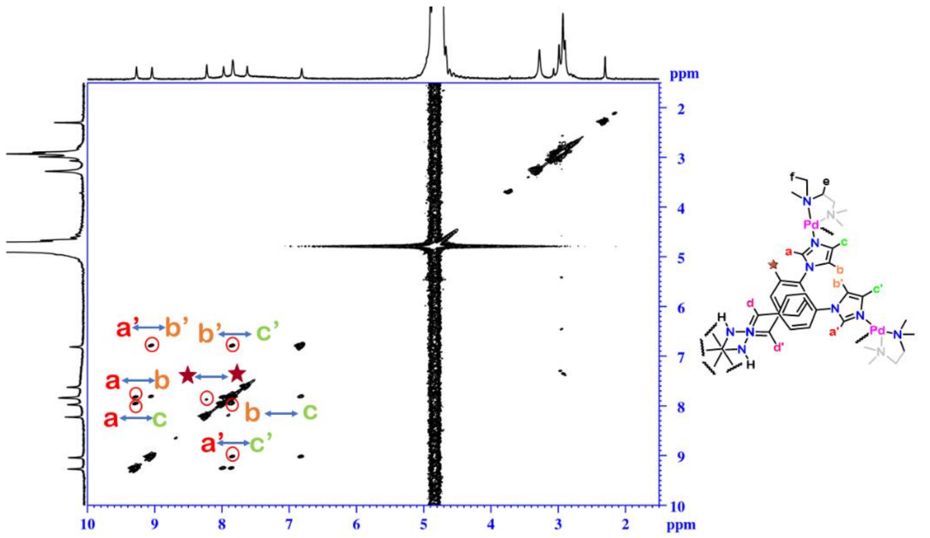

Fig. S18: ^{1}H-^{1}H COSY NMR spectrum of **1** in D_2O.

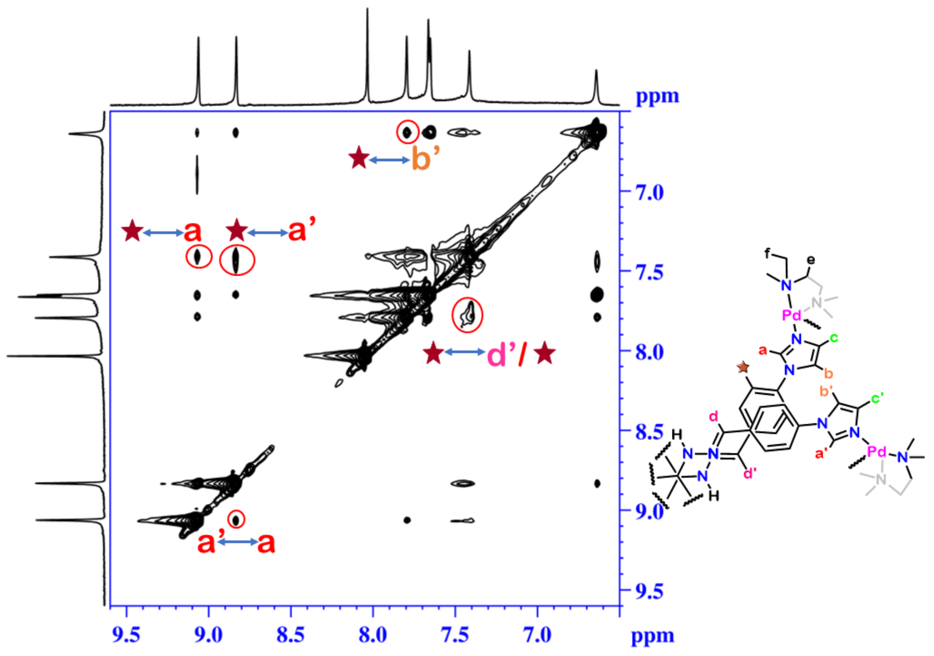

Fig. S19: Partial ^{1}H-^{1}H NOESY NMR spectrum of **1** in D_2O.

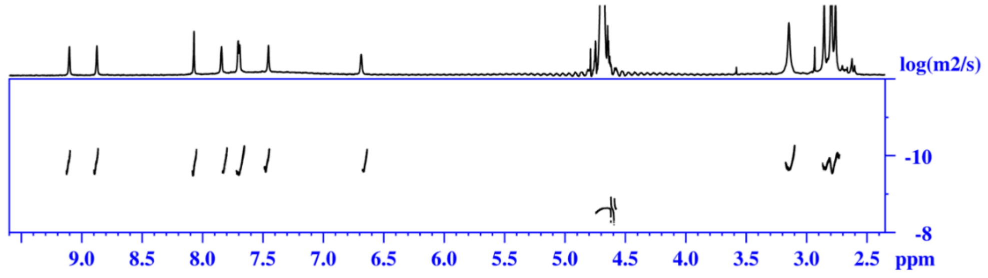

Fig. S20: ^{1}H DOSY NMR spectrum of **1** in D_2O.

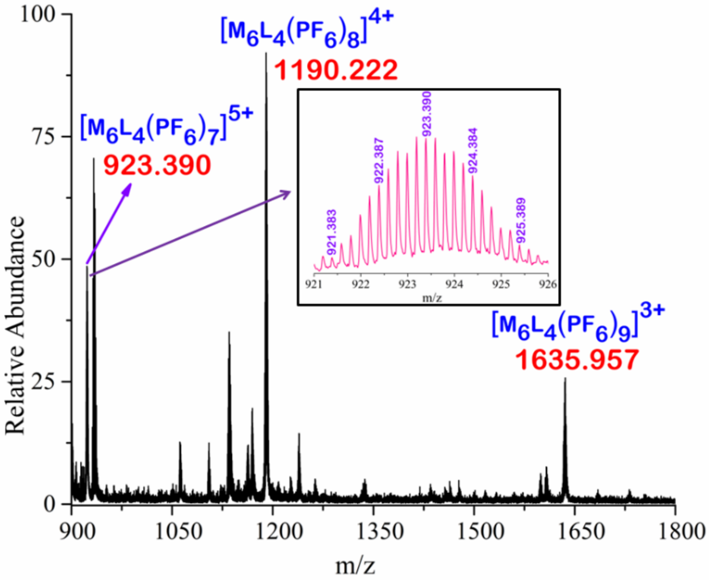

Fig. S21: ESI-MS spectrum of the PF_6^- analogue of **1** in CH_3CN. (Inset) Isotopic distribution pattern of the $[M_6L_4(PF_6)_7]^{5+}$ fragment.

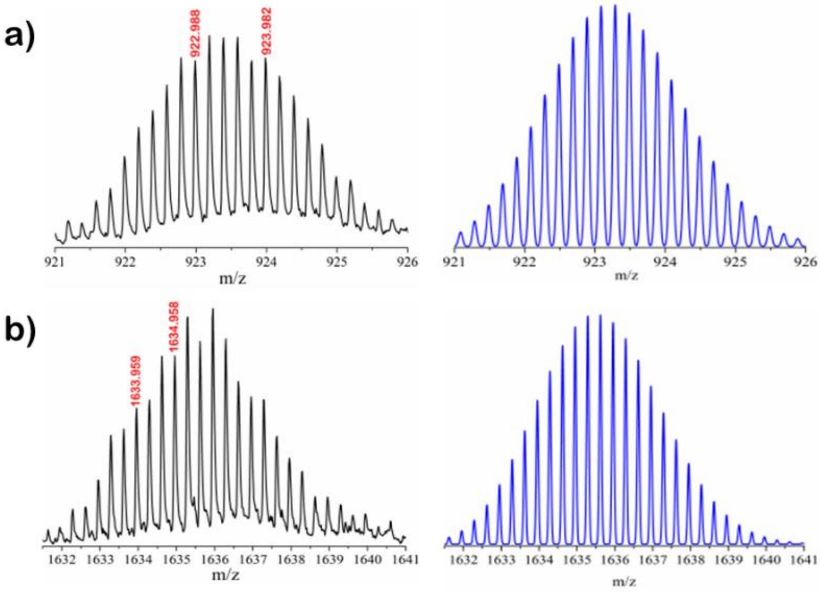

Fig. S22: Isotopic distribution patterns of the peaks corresponding to (a) $[M_6L_4(PF_6)_7]^{5+}$ (Black- experimental, blue- calculated) and (b) $[M_6L_4(PF_6)_9]^{3+}$ (Black- experimental, blue-calculated) fragments.

5. Host-guest chemistry of 1:

5.1 General procedure for the formation of the host-guest complexes:

The host-guest complexes of **1** were obtained in two different ways:

(a) **In-situ complexation**: To a clean 4 mL glass vial a mixture of **M** (5.0 mg, 14.41 μmol), **L·HNO₃** (6.0 mg, 9.61 μmol) and anthracene(**A**) or phenanthrene(**P**) (or any other guest) (5.0

mg, excess) was taken followed by the addition of 0.5 mL D_2O. The mixture was then stirred for 12 hours at 55 ^oC to get an orange turbid mixture. The excess guest was removed via centrifugation. The clear orange supernatant was then isolated and characterized by ^1H NMR spectroscopy.

(b) **Complexation after the formation of the cage**: To a clean 4 mL glass vial a mixture of the acceptor **M** (5.0 mg, 14.41 µmol) and the ligand **L·HNO$_3$** (6.0 mg, 9.61 µmol) was taken followed by the addition of 0.5 mL D_2O. The mixture was then stirred for 12 hours at 55 ^oC to get a turbid orange solution. The solution was centrifuged to get the clear solution of the interlocked cage **1**. The solution was then mixed with the guest (5 mg, excess) and stirred for overnight. The orange solution was then isolated by centrifugation and characterized by ^1H NMR spectroscopy.

The ^1H NMR spectra obtained from both the methods were identical and hence any one of the methods could be used to obtain host-guest complexes.

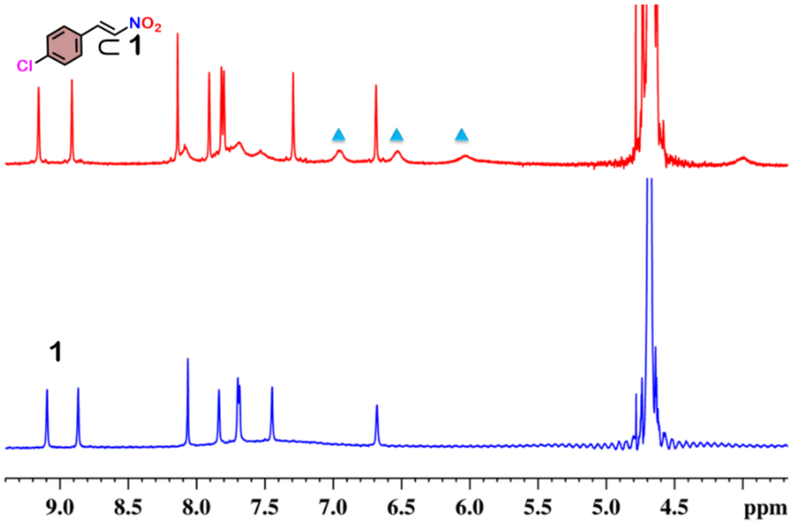

Fig. S23: ^1H stack plot of **1** and **Ns⊂1** [Ns: (E)-1-chloro-4-(2-nitrovinyl)benzene)]. (▲denotes guest peaks).

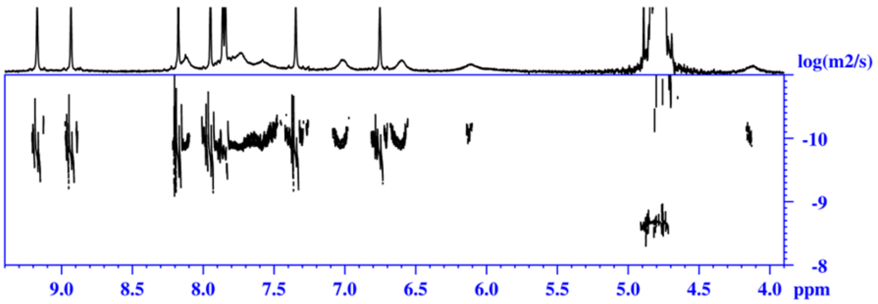

Fig. S24: ^1H DOSY NMR spectrum of **Ns⊂1** in D_2O.

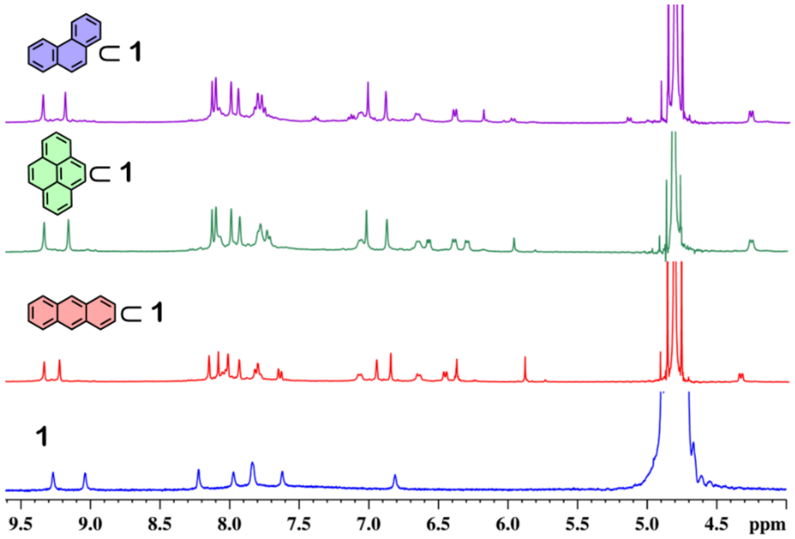

Fig. S25: ^{1}H stack plot of **1**, **A**⊂**1** (**A**: anthracene), pyrene⊂**1**, and **P**⊂**1** (**P**: phenanthrene) in D_2O showing the change in NMR by guest encapsulation (Inset).

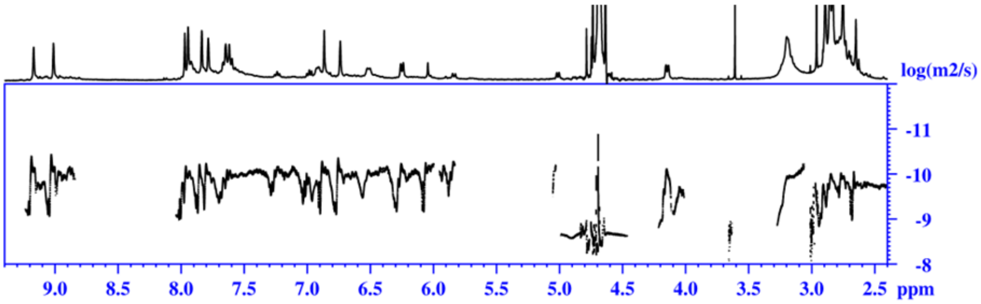

Fig. S26: ^{1}H DOSY NMR spectrum of **P**⊂**1** (**P**: phenanthrene) in D_2O.

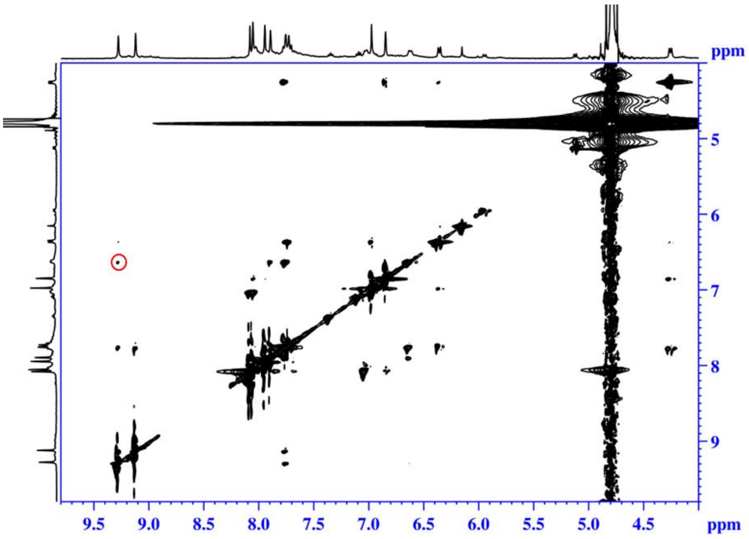

Fig. S27: Partial ^{1}H-^{1}H NOESY NMR spectrum of **P**⊂**1** (**P**: phenanthrene) in D_2O. Red mark indicates the interaction between host and guest peak.

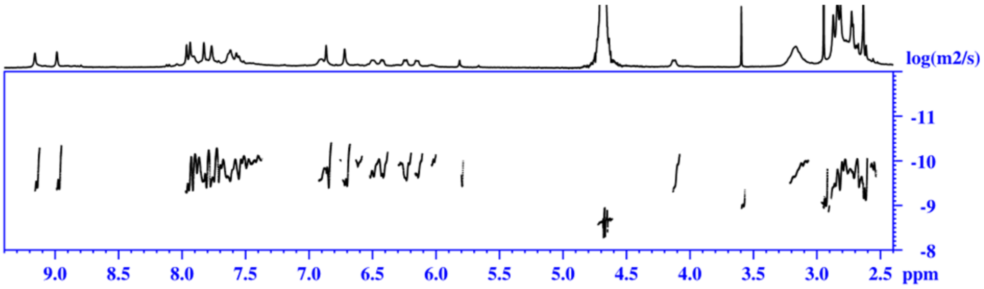

Fig. S28: ^{1}H DOSY NMR spectrum of **A⊂1** (**A**: anthracene) in D_2O.

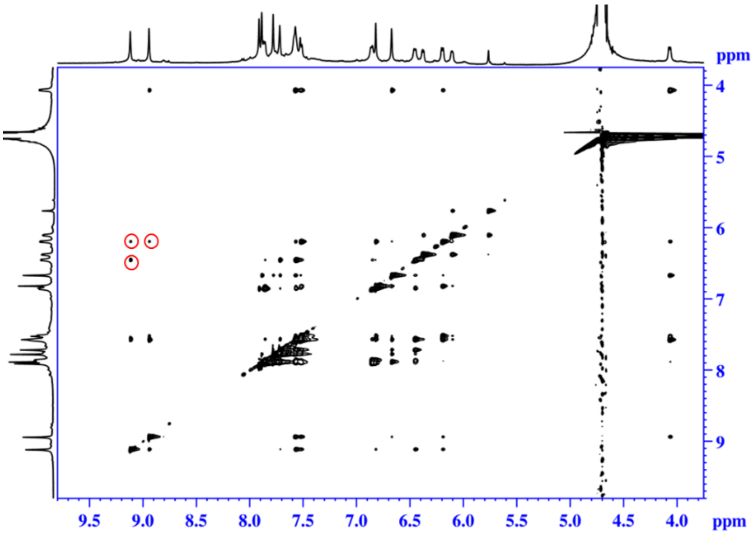

Fig. S29: Partial ^{1}H-^{1}H NOESY NMR spectrum of **A⊂1** (**A**: anthracene) in D_2O. Red mark indicates the interaction between host and guest peak.

5.2 Determination of apparent association constant of 1 with different guests:

The binding behavior of capsule **1** was studied by competitive ^{1}H NMR titration of **1** with anthracene (**A**) and phenanthrene (**P**). Titration experiments were carried out in D_2O. The D_2O stock solution of **1** with an internal standard of tetrabutylammonium nitrate [$(N(Bu)_4)NO_3$] was prepared and stock solutions of **A** in DMSO- d_6 (0.017 M) and **P** in CD_3OD (0.017 M) were also prepared. The ^{1}H NMR titration was performed by adding aliquots of guest stock solution to 0.5 mL of **1** stock solution. It was found that the guests (**A** and **P**) were bound to **1** by slow exchange on the NMR time scale at 25 °C. Thus, the host-guest concentration [HG] could be calculated by integration of $H_{a'}$ proton with respect to the internal standard.

The binding constant (K_a) and Hill coefficient value (n) were calculated using the Hill equation.[7]

$$\theta = \frac{[HG_n]}{[HG_n] + [H]} \quad \text{(S1)}$$

$$log(\frac{\theta}{1-\theta}) = nlog[G] + nlog[K_a] \quad \text{(S2)}$$

$$[H]_0 = [H] + [HG] \quad \text{(S3)}$$

$$[G]_0 = [G] + [HG] \quad \text{(S4)}$$

Where θ is the fraction of binding sites (host molecule) occupied by the guest, [G] is the guest concentration, n is the Hill coefficient describing cooperativity, and K_a is the apparent association constant.

In the Hill equation, the value of θ was obtained using equation S1. The integration of the NMR peak of $H_{a'}$, gave the concentrations of the host-guest complex [HG]. Knowing the concentration of [HG], the concentration of guest [G] and that of host [H] can be calculated from the mass balance equation S3 and S4. With the value of θ and [G] calculated, a linear plot can be obtained using the equation S2. From the slope and intercept of such plot n and K_a can be calculated.

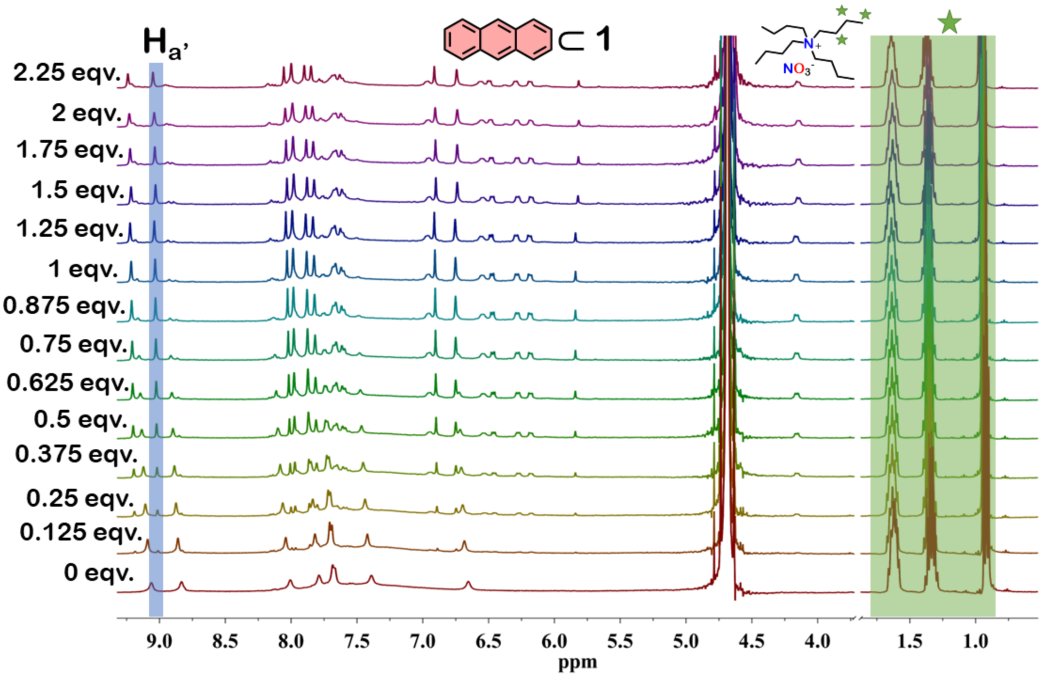

Fig. S30: ^{1}H NMR titration of **1** (c = 3.65 mM) upon addition of 0 – 2.25 eqv. of **A** (anthracene). Imidazole proton $H_{a'}$ are highlighted in the blue box. The peaks of the internal standard tetrabutylammonium nitrate are indicated by ★.

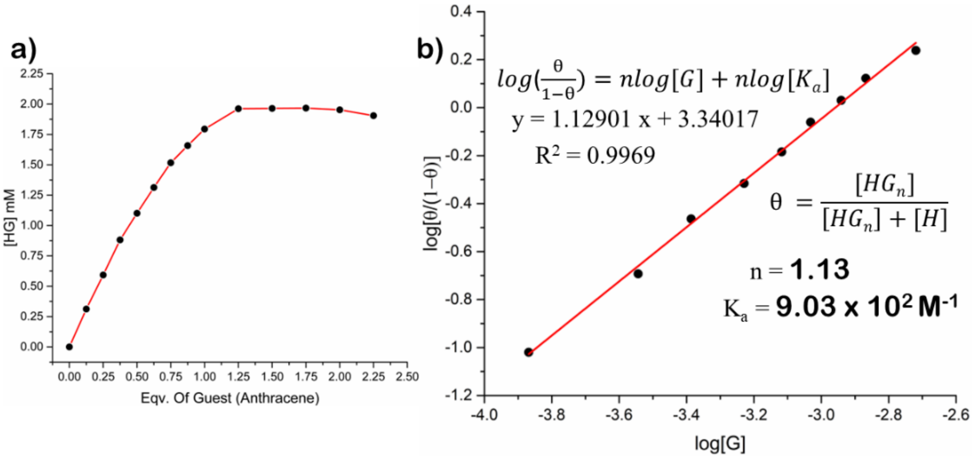

Fig. S31: Titration curve of **1** with **A**(Anthracene) a) plot of host-guest concentration [HG] vs equivalence of guest added b) plot of $log(\frac{\theta}{1-\theta})$ vs log[G].

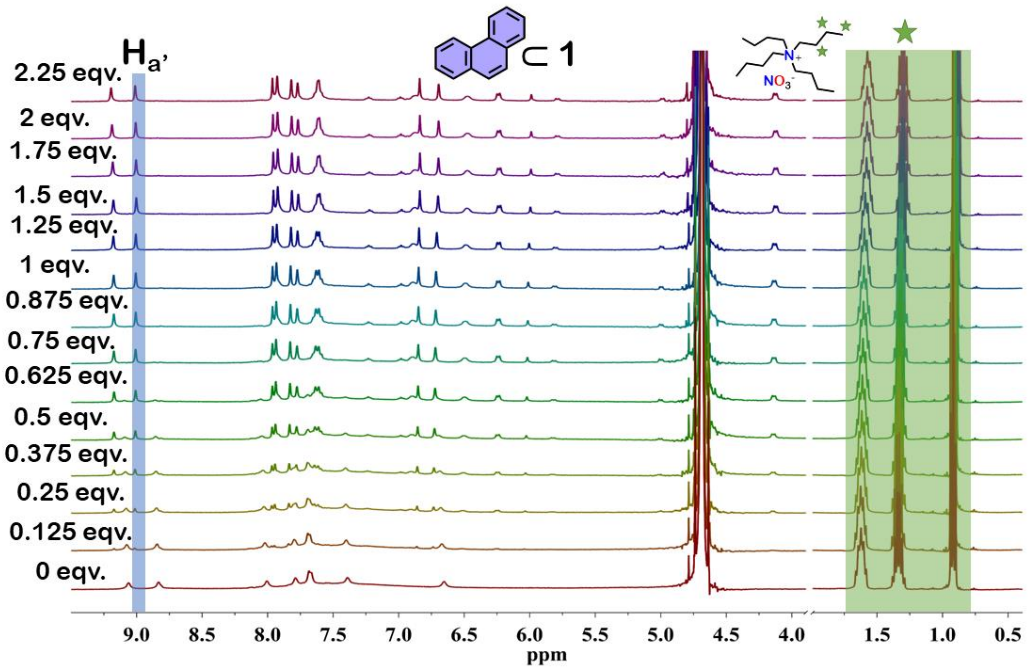

Fig. S32: ^{1}H NMR titration of **1** (c = 2.53 mM) upon addition of 0 – 2.25 eqv. of **P** (phenanthrene). Imidazole proton $H_{a'}$ are highlighted in the blue box. The peaks of the internal standard Tetrabutylammonium nitrate are indicated by ★.

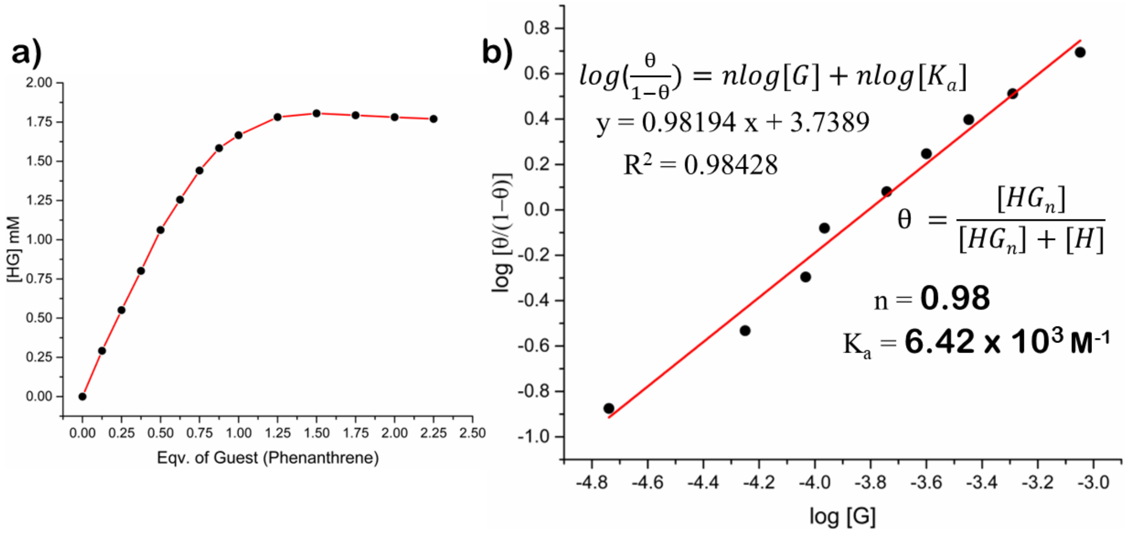

Fig. S33: Titration curve of **1** with **P**(Phenanthrene) a) plot of host-guest concentration [HG] vs equivalence of guest added b) plot of $log(\frac{\theta}{1-\theta})$ vs log[G].

6. Spectral characterization of 2:

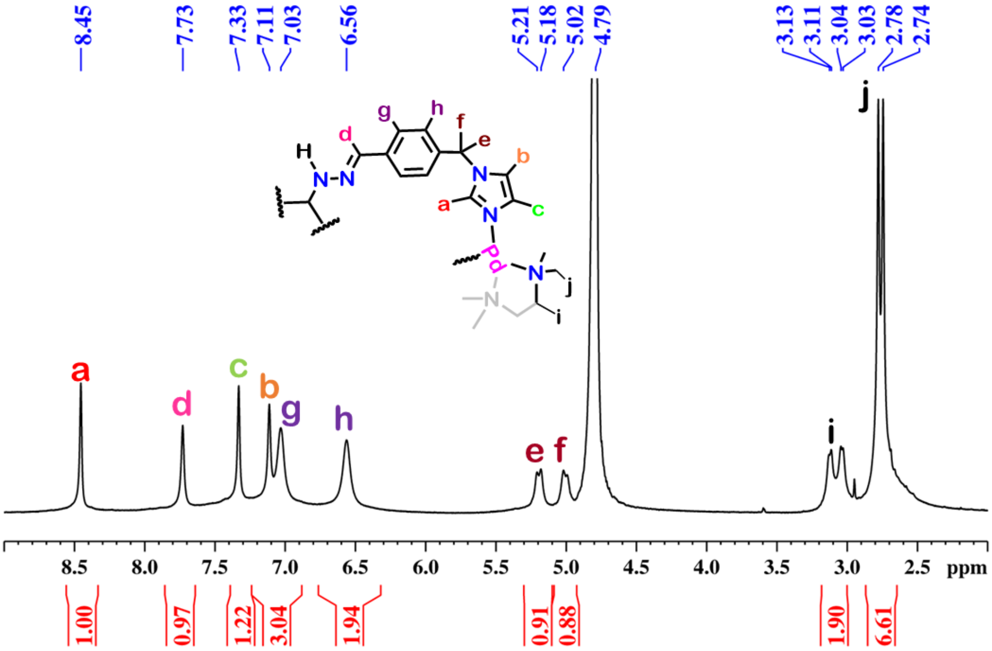

Fig. S34: ^{1}H NMR spectrum of **2** in D_2O.

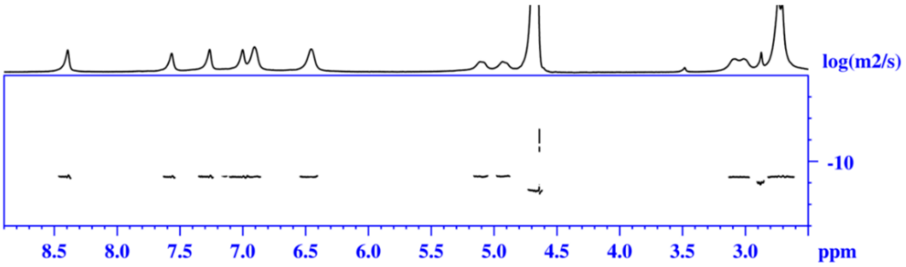

Fig. S35: ^{1}H DOSY NMR spectrum of **2** in D_2O.

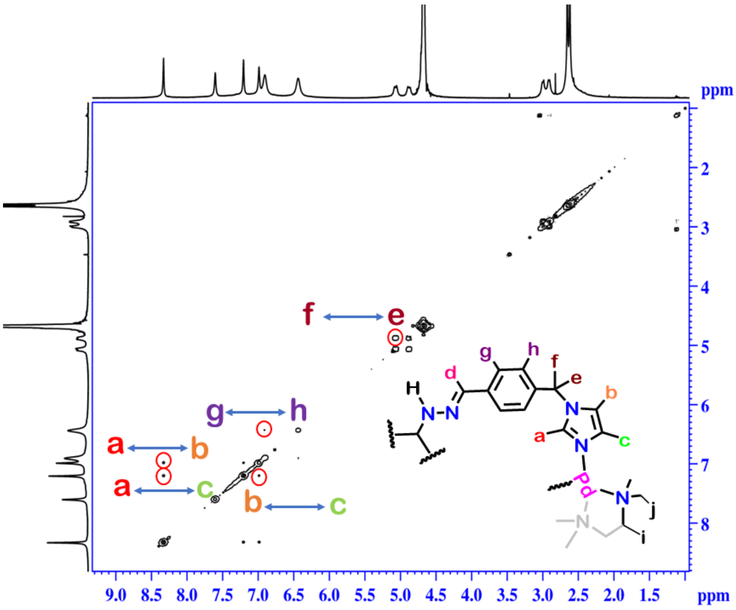

Fig. S36: ^{1}H-^{1}H COSY NMR of **2** in D_2O.

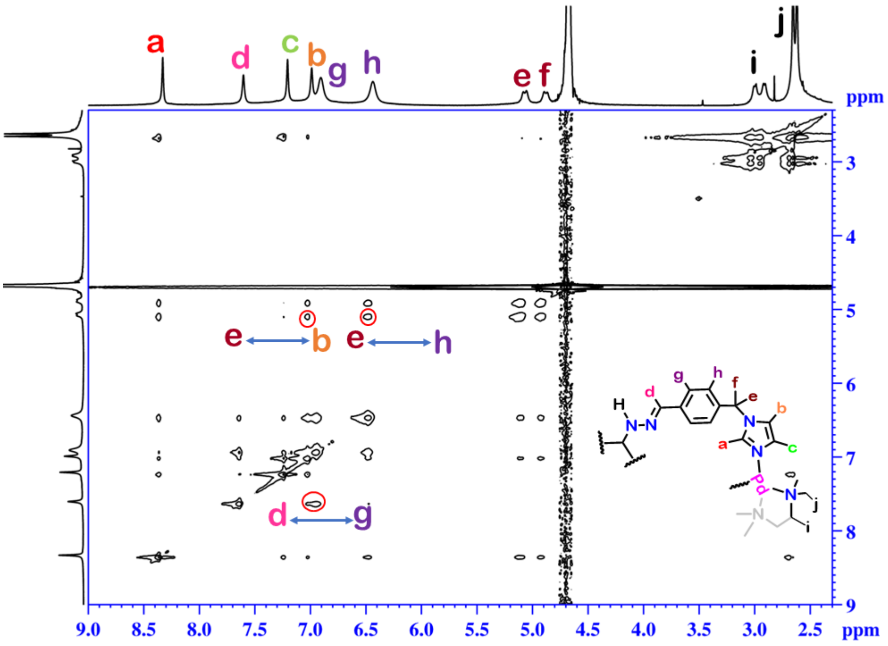

Fig. S37: ^{1}H-^{1}H NOESY NMR of **2** in D_2O.

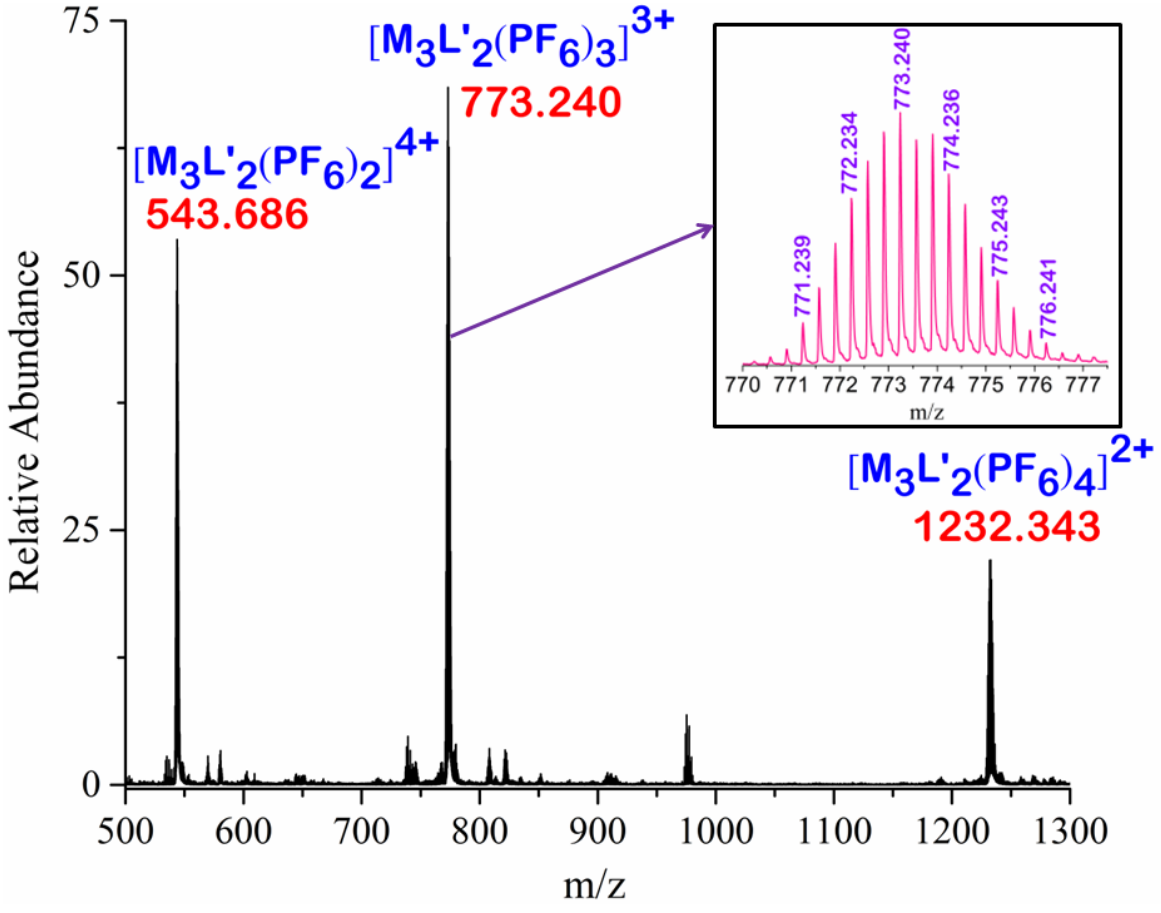

Fig. S38: ESI-MS spectrum of the PF_6^- analogue of **2** in CH_3CN. (Inset) Isotopic distribution pattern of the $[M_3L'_2(PF_6)_3]^{3+}$ fragment.

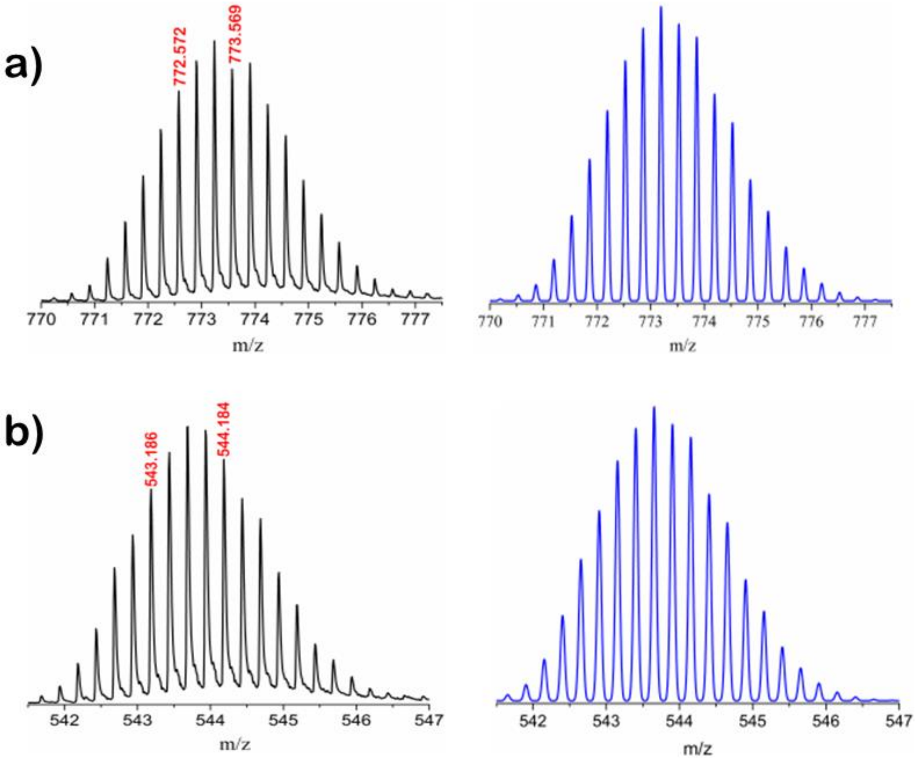

Fig. S39: Isotopic distribution patterns of the peaks corresponding to (a) $[M_3L'_2(PF_6)_3]^{3+}$ (Black- Experimental; Blue- Calculated) and (b) $[M_3L'_2(PF_6)_2]^{4+}$ (Black-experimental, blue-calculated) fragments.

7. Selective host-guest chemistry:

To check selective host-guest chemistry of **1,** the complex was prepared as follows:

To a clean 4 mL glass vial a mixture of the acceptor **M** (5 mg, 14.41 µmol), and the ligand **L·HNO₃** (6.05 mg, 9.61 µmol) was taken followed by the addition of 0.5 mL D_2O. The mixture was then stirred for 12 hours at 55 ^{o}C to get a turbid orange solution. The solution was centrifuged to get the clear solution of the interlocked cage **1**. To this solution was added a solid mixture of equivalent amount of planar guest (5 eqv. of **L·HNO₃**) (**A** or **P**) and non-planar guest (5 eqv. of **L·HNO₃**) (**H₂A:** 9,10-dihydroanthracene or **S₂A**: thianthrene). This mixture was then stirred for 12 hours at room temperature. This solution was then centrifuged to obtain the host-guest complex.

To extract the guest from the host-guest complex, the D_2O solution of the host-guest complex was stirred with 0.5 mL of $CDCl_3$ overnight. This solution was then centrifuged and the $CDCl_3$ part was taken for recording ^{1}H NMR.

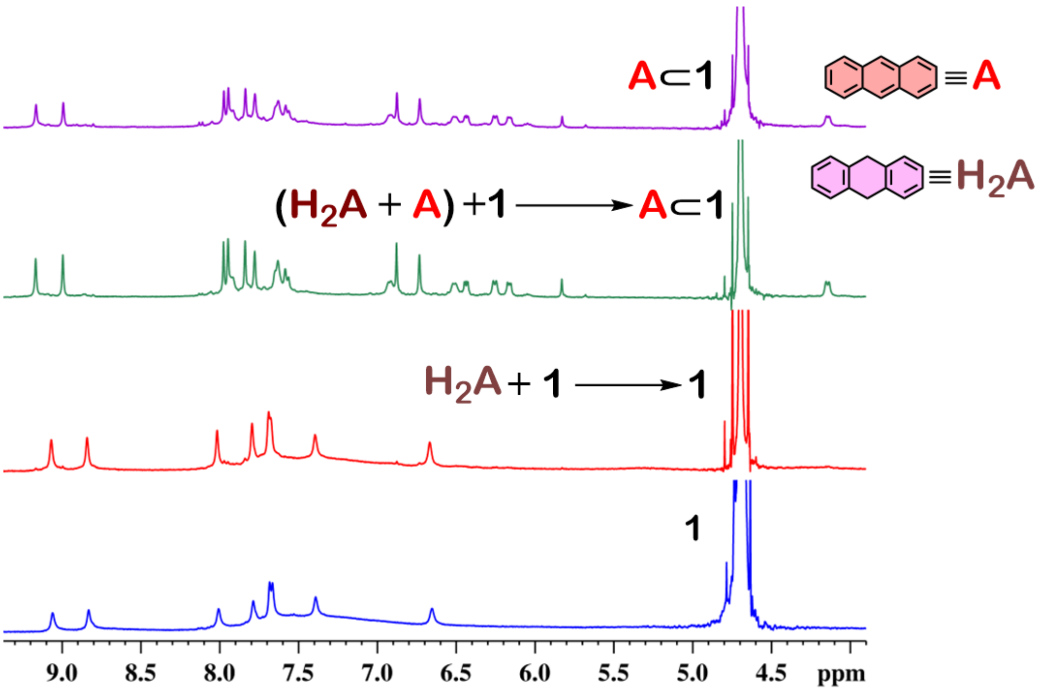

Fig. S40: ^{1}H stack plot of **1**, **H_2A**+**1** (**H_2A**: 9,10-dihydroanthracene), (**A**+**H_2A**)⊂**1** and **A**⊂**1** (**A**: Anthracene) in D_2O showing the change in NMR by guest encapsulation.

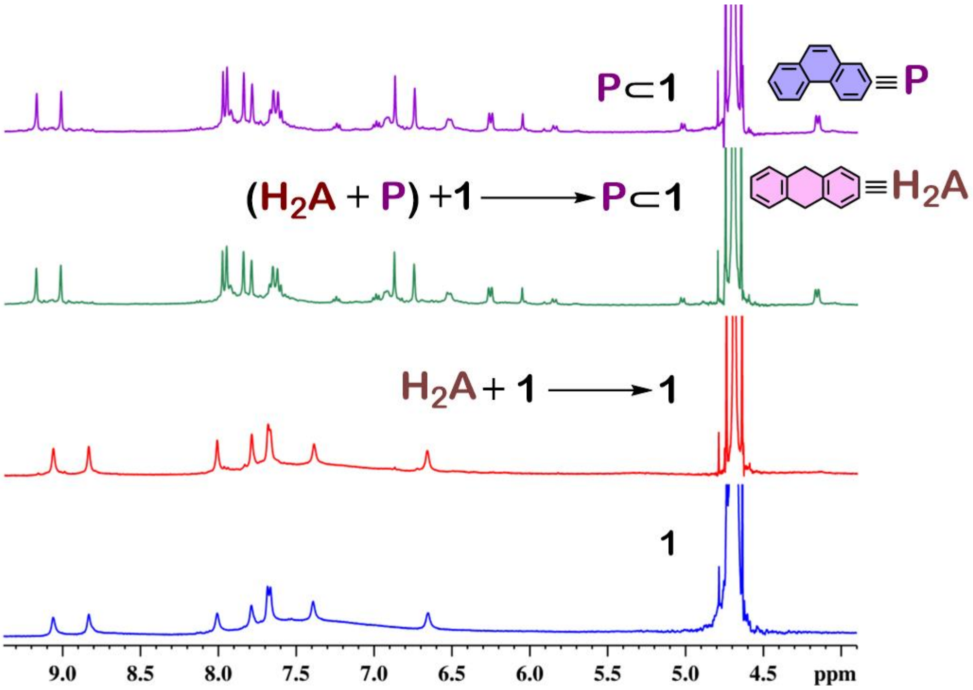

Fig. S41: ^{1}H stack plot of **1**, **H_2A**+**1** (**H_2A**: 9,10-dihydroanthracene), (**P**+**H_2A**)⊂**1** and **P**⊂**1** (**P**: phenanthrene) in D_2O showing the change in NMR by guest encapsulation.

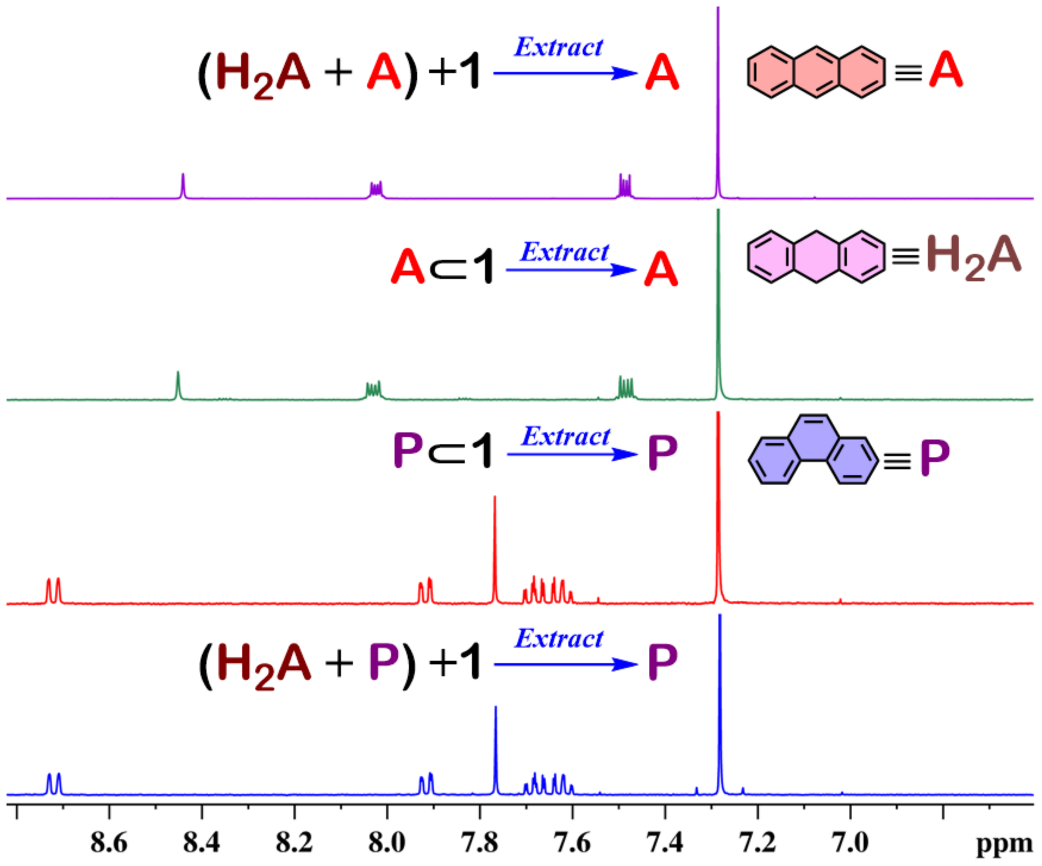

Fig. S42: ^{1}H NMR stack plot of the solid obtained after extracting the dissolved guest from (**P**+**H₂A**)⊂**1**, **P**⊂**1**, **A**⊂**1** and (**A**+**H₂A**)⊂**1** in $CDCl_3$.

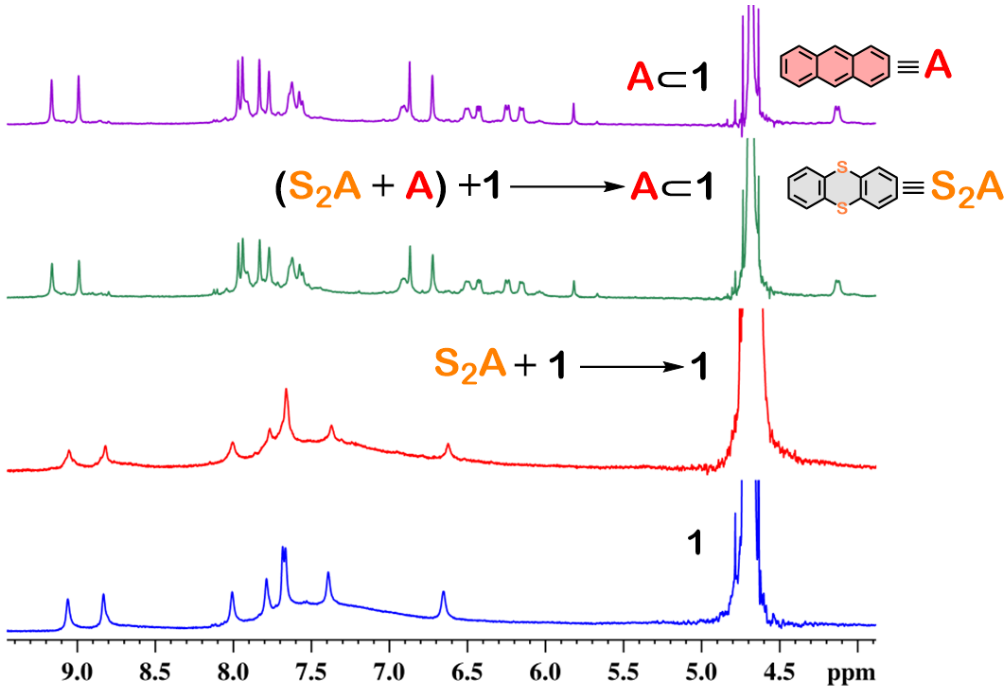

Fig. S43: Partial ^{1}H stack plot of **1**, **S₂A**⊂**1** (**S₂A**: thianthrene), (**A**+**S₂A**)⊂**1** and **A**⊂**1** in D_2O showing the change in NMR by guest encapsulation.

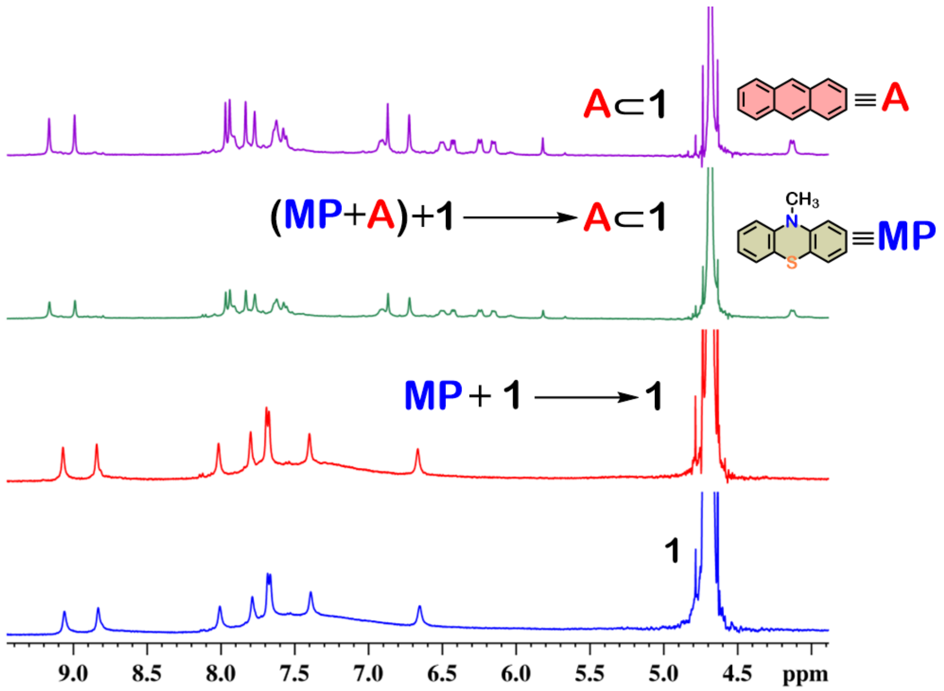

Fig. S44: Partial ^{1}H stack plot of **1**, **MP⊂1** (**MP**: N-Methyl phenothiazine), (**A**+**MP**)⊂**1** and **A⊂1** in D_2O showing the change in NMR by guest encapsulation.

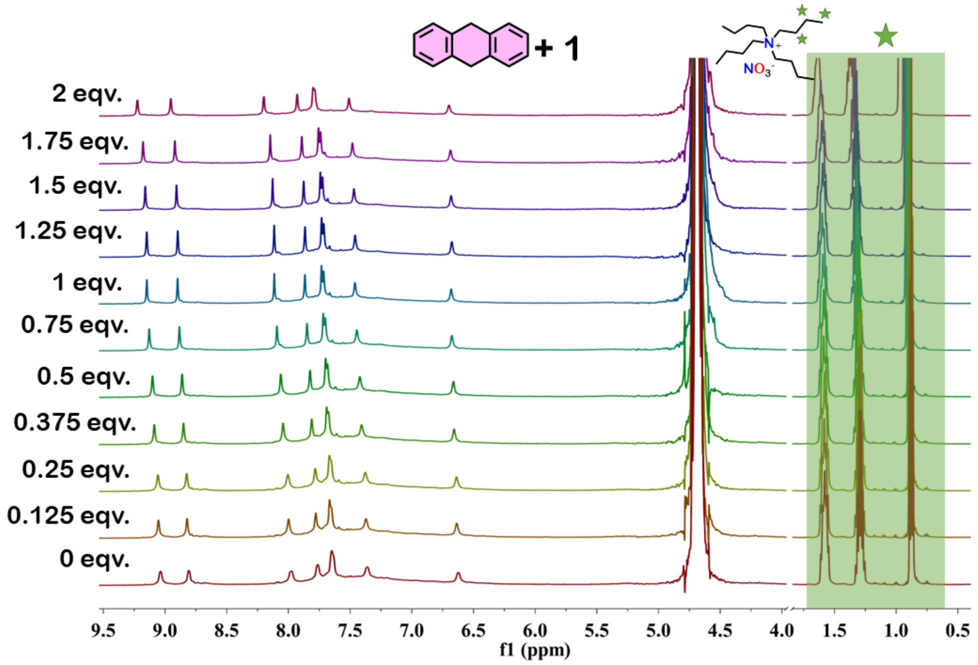

Fig. S45: ^{1}H NMR titration of **1** (c = 1.82 mM) upon addition of 0 – 2 eqv. of **H_2A** (9,10-dihydroanthracene). The peaks of the internal standard tetrabutylammonium nitrate are indicated by ★. This titration shows no change in ^{1}H NMR as **1** did not encapsulate **H_2A**.

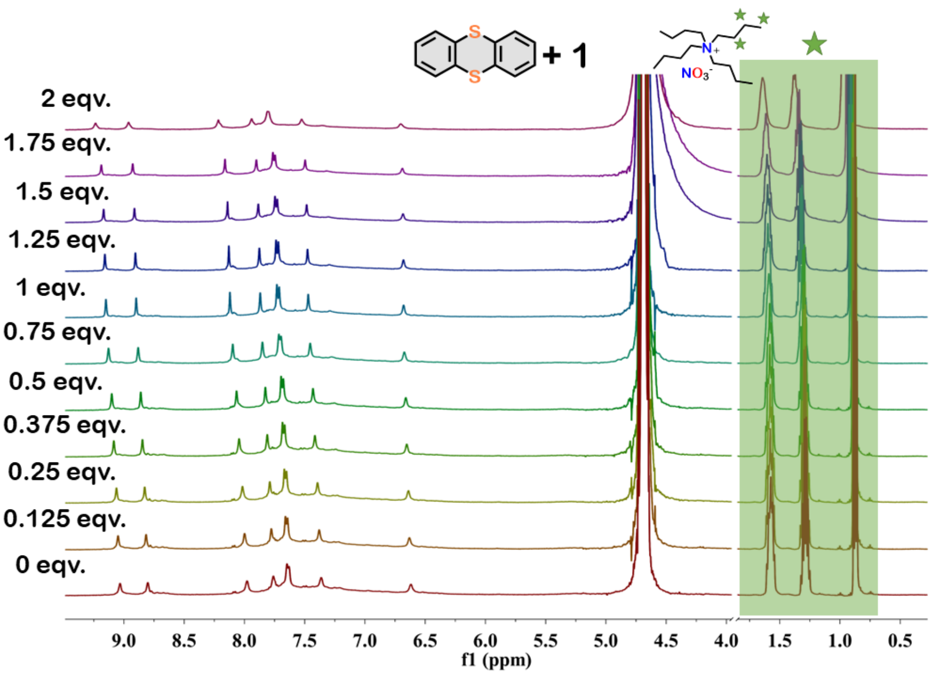

Fig. S46: ^{1}H NMR titration of **1** (c = 1.69 mM) upon addition of 0 – 2 eqv. of **S2A** (N-Methyl phenothiazine). The peaks of the internal standard tetrabutylammonium nitrate are indicated by★. This titration shows no change in ^{1}H NMR as **1** did not encapsulate **S2A**.

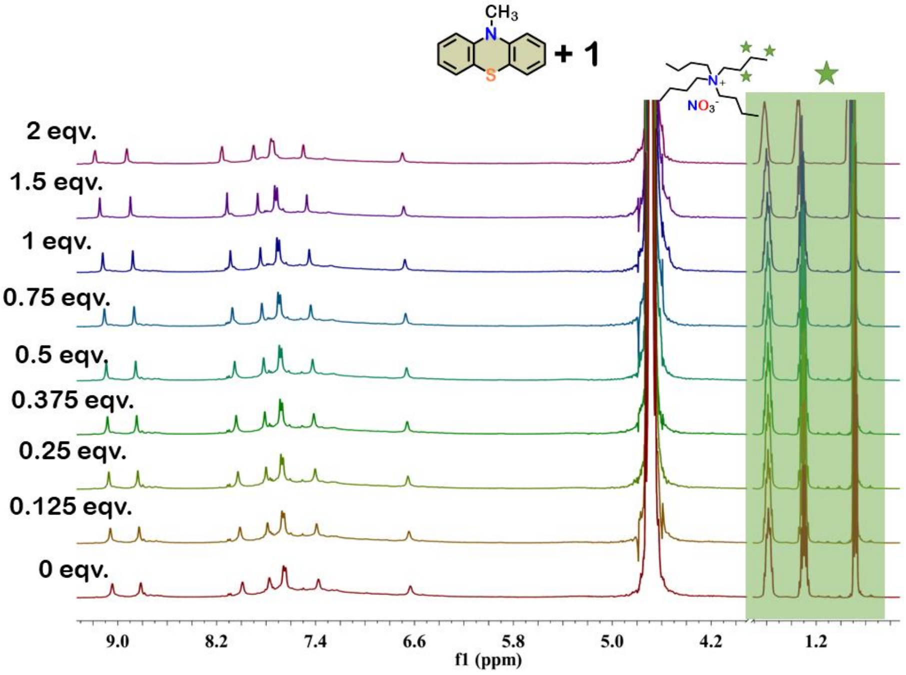

Fig. S47: ^{1}H NMR titration of **1** (c = 2.01 mM) upon addition of 0 – 2 eqv. of **MP** (thianthrene). The peaks of the internal standard tetrabutylammonium nitrate are indicated by★. This titration shows no change in ^{1}H NMR as **1** did not encapsulate **MP**.

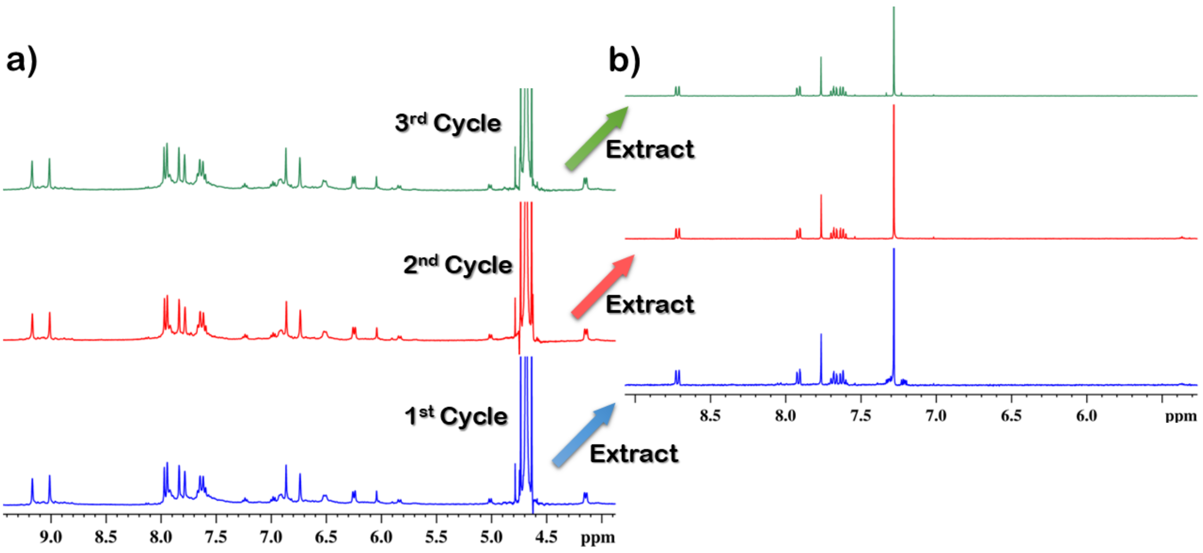

Fig. S48: Reproducibility check a) Partial ^{1}H stack plot of (**P**+**H$_2$A**)⊂**1** in D_2O , in each cycle equimolar amount of **P** and **H$_2$A** was added, b) Partial ^{1}H stack plot of the extracted solid in $CDCl_3$ from each cycle, showing the presence of only peaks for **P**.

8. Optimized structures

All the computational studies were carried out using Gaussian 09 package.[8] Full geometry of the inclusion complex **A⊂1** was optimized using semiempirical method with PM6 basis set. The structure of 1 as obtained from the SCXRD was used as a starting point. As the number of atoms were high, initial optimization was done by fixing the coordinated of the host and only optimizing the guest (A) inside the host (1). Then, the whole structure was optimized by unfreezing the coordinates of the host. In all calculations, solvation was considered introducing Polarization Continuum model (PCM) choosing water as a solvent for better comparison with experimental results.

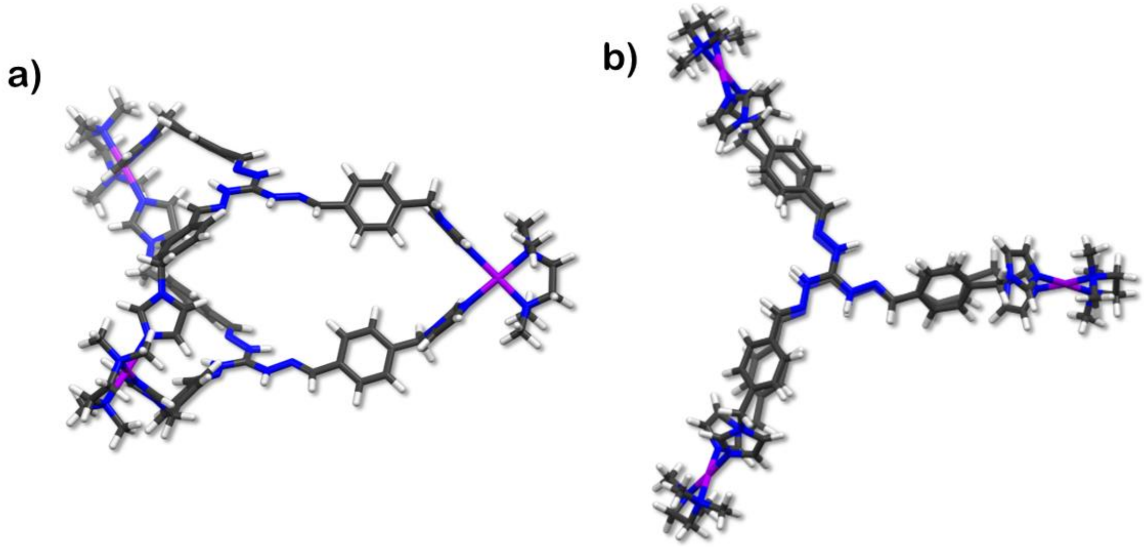

Fig. S49: Optimised structure of **2** (PM6 level), a) Side view, (b) Top view (capped-stick model) [Colour scheme: H, white; C, black; N, blue; Pd, violet]

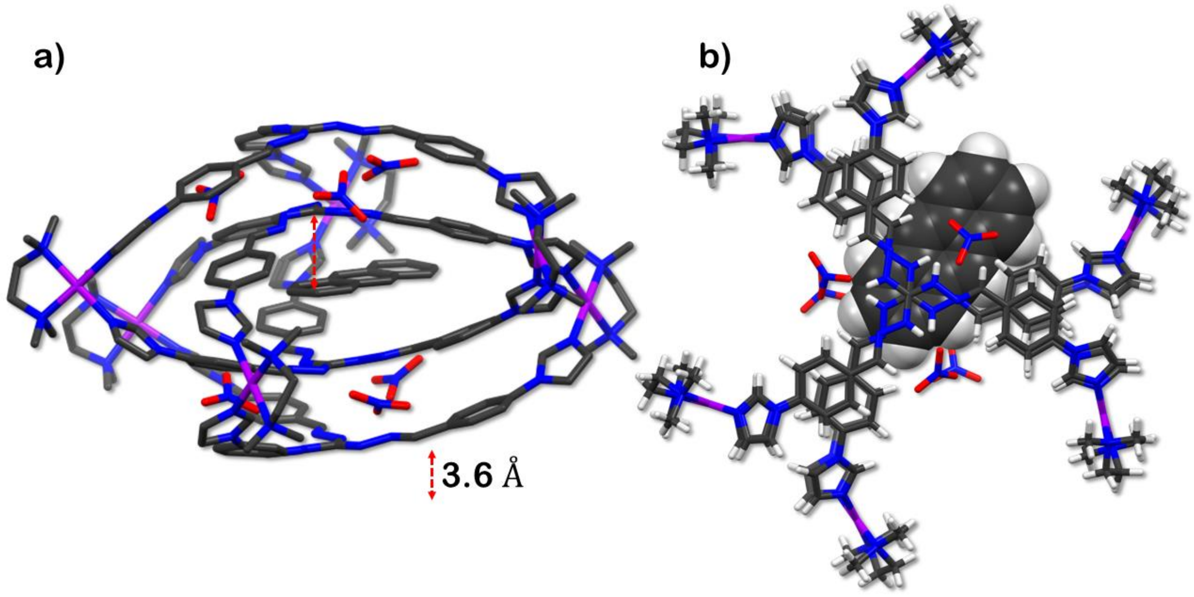

Fig. S50: Optimised structure of **A**⊂**1** (PM6 level), a) Side view, (b) Top view [Colour scheme: H, white; C, black; N, blue; O, red; Pd, violet]

Coordinates of **2**:

N	6.74897300	-8.62998200	1.56086100
N	4.40741100	10.08144700	-1.35859200
N	6.19345400	-7.29125700	3.30568700
N	6.86873500	-8.60804400	-1.43982100
N	5.84726600	-7.56197400	-3.17231100
N	3.10931100	9.12365800	3.23376700
N	3.88465900	10.32048500	1.47480100
N	3.75301100	8.74663700	-3.06840300
C	6.06255300	-8.56544700	2.73812300
H	5.46646700	-9.36161000	3.16936200
C	3.33052500	-6.20928800	3.90431300
H	3.24510900	-7.15516200	3.36671900
C	2.36593900	4.18225000	4.63008600
C	3.52820400	-3.77967700	5.28677100
H	3.61247300	-2.83891500	5.83590000
N	-10.95587500	-1.32486000	-1.41138700
C	2.31246900	-4.14540700	4.69044500

C	2.55165500	4.05600100	-4.84277300
C	2.22728800	-5.36044600	3.98097000
H	1.29328800	-5.65533300	3.50010200
C	4.53291000	-5.86641300	4.54366700
C	6.99445600	-6.53192600	2.44723500
H	7.27556500	-5.50919800	2.64449000
C	4.63084700	-4.63812500	5.21247200
H	5.56252900	-4.34911200	5.70811600
C	3.63280000	6.08836500	3.80739300
H	4.48406300	6.48214900	3.24982600
C	2.25190300	-4.27212800	-4.88679500
C	3.44355800	4.70936500	3.89038800
H	4.15038300	4.04333400	3.39355100
C	2.75764800	6.96356400	4.47028800
C	7.07314300	-7.85774700	-2.56092100
H	8.03306500	-7.50927000	-2.92562600
C	1.65966800	6.44078200	5.16831800
H	0.96216800	7.10725000	5.68456200
C	7.32800100	-7.36772600	1.37865200
H	7.93950300	-7.14022700	0.51415000
C	4.45777500	-5.98609500	-4.55628600
C	2.91826100	6.81490600	-4.44155300
C	3.19399600	4.83473200	-5.82161400
H	3.55194300	4.38032600	-6.74932500
C	3.59381900	-6.14721800	-5.65150100
H	3.77337500	-6.93486100	-6.39275900
C	1.46165600	5.05779300	5.24897700
H	0.61891100	4.66550700	5.82332200
C	4.24800600	9.64977700	2.60452800

H	5.25836900	9.51664400	2.97115200
C	2.49716900	-5.30036500	-5.81638800
H	1.83966800	-5.44223300	-6.67921100
C	2.09298200	4.66509700	-3.66767600
H	1.58623600	4.06891200	-2.91041000
C	3.48473400	9.92291700	-2.35231800
H	2.65804800	10.58954500	-2.56753200
C	4.84382100	-8.16247500	-2.40860600
H	3.79640800	-8.11514700	-2.66151400
C	3.37107500	6.20466700	-5.62139600
H	3.86307700	6.79606000	-6.40147200
C	-4.79173000	0.15651700	-4.84368700
C	5.48619100	-8.79221100	-1.33779400
H	5.04721100	-9.36384900	-0.52932800
C	5.67701400	-6.85134500	4.61898200
H	6.53217700	-6.42187500	5.20610500
H	5.34477600	-7.74539700	5.21058900
C	2.28247700	6.03587400	-3.46624200
H	1.92062200	6.50022300	-2.55212600
C	4.20758600	-4.96697400	-3.62732100
H	4.87315100	-4.83165800	-2.77692400
C	5.68713900	-6.86021800	-4.46793800
H	5.67979000	-7.64069100	-5.27807300
H	6.59315200	-6.23217600	-4.66975700
C	3.11494200	-4.11013900	-3.79411800
H	2.93437900	-3.31325500	-3.07343300
C	2.00175900	9.47599700	2.45805200
H	0.98601700	9.21418400	2.71525000
C	5.26987100	8.98786300	-1.43851100

H	6.09995700	8.87101900	-0.75584000
C	3.04850000	8.31414200	-4.29962000
H	3.60051400	8.75114100	-5.17894900
H	2.02783000	8.77603300	-4.33261100
C	2.49382400	10.20963900	1.37632400
H	1.95182200	10.65797600	0.55613000
C	4.88609200	8.16226100	-2.50095400
H	5.33264900	7.25089300	-2.86751500
C	3.05490500	8.44411800	4.54529300
H	2.29186900	8.96844600	5.17982700
H	4.02434600	8.59496800	5.08815300
N	-9.47300600	-1.16915100	-3.11859100
C	-5.78322200	0.35552900	-5.82055400
H	-5.56188500	0.91381800	-6.73434400
C	-10.42213400	-0.03501900	-1.43747700
H	-10.72587500	0.71594300	-0.72114100
C	-5.09813500	-0.57422500	-3.68801200
H	-4.33160000	-0.74155600	-2.93306300
C	-7.37290500	-0.89735000	-4.47661100
C	-9.51436800	0.07908300	-2.49644400
H	-8.93744800	0.92878500	-2.82840100
C	-10.36597600	-2.00834400	-2.43536000
H	-10.53840400	-3.04729600	-2.69158200
C	-7.06226400	-0.17171900	-5.63719300
H	-7.81718000	-0.01652800	-6.41582900
C	-6.38435800	-1.09193000	-3.50339500
H	-6.61156900	-1.66121600	-2.60474800
C	-8.74186200	-1.52638300	-4.35865100
H	-8.64049200	-2.63988400	-4.42747900

H	-9.38980700	-1.23205100	-5.23014900
N	-0.06837000	-1.40888800	-5.08740200
H	-1.00190000	-1.85757300	-5.18782200
N	1.10796300	-2.13818000	-4.90921000
C	0.01021700	-0.01872800	-5.06432200
C	1.04980600	-3.43157600	-5.08218600
H	0.14494900	-4.00203900	-5.38471300
N	-1.15559500	0.74502700	-5.07845000
H	-1.07879100	1.77689200	-5.19019000
N	-2.37256900	0.09134400	-4.88559900
C	-3.46663100	0.78243500	-5.05693400
H	-3.51832000	1.84682100	-5.37181500
N	1.25365100	0.61031900	-5.09229000
H	2.10859300	0.02882900	-5.21126400
N	1.29482900	1.99044400	-4.89599600
C	2.43671900	2.59742600	-5.07215900
H	3.38625600	2.11551100	-5.39108100
N	1.14902100	0.71454700	4.98053700
H	2.02627300	0.16808100	5.09873700
N	1.13441600	2.10011800	4.82261400
C	-0.06744800	0.03599100	4.98114000
C	2.28751300	2.71307600	4.79108600
H	3.27959000	2.22296800	4.88544600
N	-0.08717700	-1.35636600	4.99750400
H	-0.99994700	-1.84287200	5.10606500
N	1.12236300	-2.03667600	4.85415300
C	1.07903600	-3.34221600	4.83877700
H	0.15874900	-3.95589100	4.93850600
N	-1.26385400	0.74997400	4.97355400

H	-1.23025800	1.78394600	5.07953200
N	-2.45470800	0.04103500	4.81976000
C	-15.14694500	-2.48591800	0.60788300
C	-15.23438900	-1.60921100	-0.64575000
H	-15.10402000	-3.56941800	0.33332800
H	-16.08749400	-2.38090100	1.21119500
H	-16.12214700	-1.91371700	-1.25900700
H	-15.42780300	-0.54221500	-0.37080600
C	9.62909600	-11.97540200	0.84006700
C	9.27547600	-12.25614500	-0.65817000
H	10.60945800	-11.39249300	0.92319200
H	9.81909100	-12.95072800	1.38170000
H	10.16328400	-12.72281000	-1.18509300
H	8.43801800	-13.03018900	-0.73964100
C	6.29369100	13.92996600	-0.57016400
C	5.45383500	14.30182500	0.65361500
H	7.30578300	13.56446800	-0.26200200
H	6.49616200	14.84779500	-1.18170400
H	5.99247700	15.07647800	1.26219100
H	4.49987800	14.79878000	0.34334100
Pd	4.77572300	11.57442100	0.05028300
Pd	7.78394300	-9.90094000	0.06600300
Pd	-12.44120400	-1.72193900	-0.00274300
C	6.57426700	12.14195300	-2.23307200
H	6.06960100	11.43497700	-2.91504500
H	7.17331100	12.82234100	-2.87291300
H	7.28455000	11.56261000	-1.61125800
C	4.47876400	13.43644800	-2.18400700
H	4.82978900	14.13496600	-2.97190000

H	3.91056600	12.63736400	-2.69028900
H	3.76877000	13.99958900	-1.54687100
C	6.31479100	12.65315600	2.28939900
H	6.57676800	13.39304200	3.07589100
H	6.11633400	11.69593000	2.79889200
H	7.21709800	12.51323100	1.66276500
C	3.93474400	13.29982700	2.30314500
H	3.74793500	12.44793500	2.98002000
H	4.01752100	14.20184700	2.94701100
H	3.03503900	13.42810900	1.66811700
C	-13.81622200	-0.53149900	-2.34344600
H	-14.70009300	-0.38385100	-2.99915200
H	-13.67799400	0.40034600	-1.75890800
H	-12.94514200	-0.63912100	-3.01310400
C	-13.88381900	-2.99083500	-2.19459600
H	-14.64870000	-3.06333200	-2.99649100
H	-12.89945300	-3.11096400	-2.67641700
H	-14.03514200	-3.86143600	-1.52519400
C	-14.15320500	-0.85323600	2.17989000
H	-14.92183400	-0.96162500	2.97513000
H	-13.22458200	-0.51874800	2.67002700
H	-14.48775800	-0.03295300	1.51541200
C	-13.53466700	-3.23548800	2.30555100
H	-12.70742900	-2.94442300	2.97417600
H	-14.36321800	-3.58092200	2.96062200
H	-13.19432900	-4.10764600	1.71251400
C	8.05765300	-11.30746800	-2.60322100
H	7.83321900	-10.39890200	-3.20144200
H	8.58808400	-11.99882100	-3.31275200

H	7.07619400	-11.80123800	-2.32674200
C	10.05696300	-10.08239300	-1.68089900
H	10.70284300	-9.90840700	-0.77118700
H	10.71720800	-10.49956100	-2.48881500
H	9.72131300	-9.08593100	-2.03453300
C	7.31291400	-12.09168800	1.85203700
H	6.96495300	-12.67255300	0.94675700
H	7.52182900	-12.83143400	2.66980000
H	6.44957700	-11.48094200	2.19662400
C	9.05033400	-10.50810400	2.76080700
H	8.23801400	-10.02175300	3.34603400
H	9.55312700	-11.20184800	3.48804800
H	9.81116600	-9.71864700	2.47661100
N	-13.93790200	-1.70664000	-1.41848400
N	-13.91227900	-2.10886800	1.39316700
N	5.14075600	13.05410500	1.44661700
N	5.58440900	12.85127500	-1.35791400
N	8.50435400	-11.19177900	1.50212200
N	8.85110900	-10.96581200	-1.33882500
C	-3.56438600	0.72869700	4.78234400
H	-3.64231100	1.83257500	4.87441700
C	-4.87223900	0.05368800	4.62546300
C	-5.86805400	0.70590100	3.87102200
C	-5.17258500	-1.16053000	5.25939600
C	-7.15194900	0.16831300	3.78556700
H	-5.64868400	1.64567100	3.36307900
C	-6.46413900	-1.69233200	5.17618300
H	-4.41096400	-1.68058000	5.84556300
C	-7.46625300	-1.02153200	4.46063500

H	-7.91882200	0.69427000	3.21501900
H	-6.68809700	-2.62481800	5.70274900
C	-8.89010300	-1.52440200	4.52613500
H	-8.95121200	-2.44902900	5.15876900
H	-9.51831800	-0.76898400	5.06560800
N	-9.49905300	-1.82509900	3.21308400
C	-9.22514100	-2.94910000	2.42962100
C	-10.52814600	-1.11216200	2.57991400
C	-10.09985200	-2.89499200	1.34192600
H	-8.48063000	-3.68771400	2.68729000
H	-10.93309600	-0.17774200	2.94965800
H	-10.19977300	-3.58438200	0.51536300
N	-10.91103100	-1.75879400	1.44281000

Coordinates of **A⊂1:**

Pd	7.72446300	7.35984900	1.81552200
N	0.84700600	2.08664600	-2.81739300
N	4.75015000	6.83247000	-1.01205800
N	6.38363200	7.44793700	0.32493800
N	-0.27675300	1.31418700	-3.05139300
H	-1.04544400	1.67025100	-3.20229300
C	3.73801300	5.99327800	-1.55386900
N	4.45123700	6.87227900	4.33559500
C	5.71981800	6.43297400	-0.19602300
H	5.90812500	5.54042300	-0.01487600
N	-0.33441900	1.30728600	6.55214900
H	-1.12278200	1.63517300	6.65296700
C	3.05694500	3.82119600	-2.32672900
H	3.26610200	2.95151500	-2.58240300
N	0.74251800	2.12240900	6.35796900

C	-0.11915100	-0.01062500	-3.05074500
C	0.50346500	3.37616800	6.25770900
H	-0.35800300	3.69333100	6.40233900
C	1.42356500	5.56955500	-2.08675700
H	0.54933700	5.87013000	-2.17652400
C	1.23461100	5.61234700	5.60965500
H	0.35824200	5.89968600	5.72844600
C	1.74310300	4.23851400	-2.35872000
N	6.15354900	7.44826400	3.07455900
C	1.57928500	4.30793000	5.93023700
C	4.81147100	8.22495500	-1.05793600
H	4.26236900	8.79013500	-1.55241600
C	2.44375300	6.46023000	-1.66709200
H	2.24441200	7.34624200	-1.47333900
N	9.34162000	7.36332700	0.57777500
C	5.51296100	6.41739600	3.63282600
H	5.75698100	5.52308400	3.54851600
C	5.81619300	8.59662600	-0.24761900
H	6.09003300	9.47058600	-0.09209700
C	4.07638300	4.67568700	-1.91607200
H	4.95910900	4.38323800	-1.88359800
C	3.84733500	4.75422900	5.35087300
H	4.73183600	4.47262600	5.28768600
C	2.19325100	6.49169300	5.11130900
H	1.96288000	7.36273400	4.88384000
C	0.64219800	3.33291400	-2.70658700
H	-0.21268500	3.67415300	-2.85015100
N	9.07558500	7.24184000	3.34792400
C	5.49723700	8.57596000	3.44904200

H	5.72412200	9.44180200	3.18633000
C	2.87835800	3.88639800	5.83370900
H	3.10737700	3.02246000	6.08777700
C	3.49756000	6.04118900	4.96562700
C	4.45334600	8.24177800	4.26698100
H	3.86727500	8.82413300	4.68886500
C	9.35588000	6.12527300	-0.29275900
H	8.55674900	6.09537500	-0.82276600
H	9.39877200	5.34359100	0.26372400
H	10.12321600	6.14709800	-0.86607200
C	10.55338900	7.39912300	1.42040300
H	11.28023200	6.95851900	0.95406800
H	10.81073400	8.32321300	1.57148800
C	9.25216500	8.56908200	4.00366800
H	9.49224000	9.22133400	3.34066300
H	9.94513400	8.50916000	4.66263300
H	8.42941600	8.82865300	4.42551400
C	-0.12664500	-0.00645900	6.57595700
C	9.36873800	8.53134600	-0.27152600
H	8.59576800	8.52955400	-0.84162800
H	10.16191000	8.51709200	-0.81370200
H	9.36289400	9.32301000	0.27081200
C	10.35278800	6.77027800	2.66623400
H	11.10807200	6.95526400	3.24503600
H	10.30793900	5.81033500	2.53312500
C	8.75353600	6.34489600	4.39127800
H	7.95300900	6.63877200	4.82652300
H	9.47351600	6.31861700	5.02355000
H	8.61750900	5.46766600	4.02676400

N	-2.41618900	-0.22148200	-2.81844200
N	-8.47915000	0.78829800	-1.01964100
N	-9.83001200	1.89705300	0.31481200
N	-1.18341900	-0.80823500	-3.05229300
H	-1.11102400	-1.65283300	-3.19950000
C	-7.24588600	0.33061300	-1.55966900
C	-8.61866600	1.82893700	-0.20511800
H	-7.93995000	2.43848800	-0.02426300
C	-5.02281600	0.82648800	-2.32938300
H	-4.37535000	1.44140200	-2.58890300
C	-5.72104400	-1.46252000	-2.08791400
H	-5.54462900	-2.37012500	-2.17794500
C	-4.72838300	-0.52119900	-2.36438300
C	-9.71575400	0.14517200	-1.06561800
H	-9.93021800	-0.61365200	-1.55924500
C	-7.00318900	-1.02378500	-1.67118000
H	-7.67098300	-1.63916400	-1.47702500
C	-10.54055600	0.83053600	-0.25686500
H	-11.43455200	0.63091400	-0.10177200
C	-6.27395100	1.28169100	-1.92259600
H	-6.46203400	2.19275200	-1.89194200
C	-3.39333100	-1.02123400	-2.70974700
H	-3.26103300	-1.93336900	-2.84795500
N	1.21419900	-1.89354400	-2.81564500
N	3.36981200	-7.64651200	-1.00664100
N	3.08407500	-9.36893000	0.32990700
N	1.10795600	-0.53358400	-3.04611800
H	1.80076100	-0.04695500	-3.20390200
C	3.15000600	-6.35045400	-1.54879200

C	2.53776100	-8.28655600	-0.19190100
H	1.67031800	-8.00331700	-0.01210500
C	1.61049500	-4.67438400	-2.32344400
H	0.75336800	-4.42092200	-2.58142400
C	3.94093100	-4.13399900	-2.07947100
H	4.63874800	-3.52740200	-2.16950300
C	2.62975600	-3.74566200	-2.35707000
C	4.54520100	-8.39593100	-1.05069100
H	5.30992300	-8.20294500	-1.54398300
C	4.20175400	-5.46309000	-1.66047500
H	5.06835600	-5.73342400	-1.46527700
C	4.36340800	-9.45177600	-0.24065700
H	4.98318300	-10.12600700	-0.08417400
C	1.84034400	-5.98469500	-1.91297600
H	1.14552900	-6.60291000	-1.88164100
C	2.39623800	-2.34064400	-2.70455600
H	3.11760000	-1.76768400	-2.84207100
N	-8.36841000	0.51680100	4.32846700
N	-1.15743100	-0.84210800	6.55303400
H	-1.04852400	-1.68844900	6.65470700
N	-2.40202400	-0.31746500	6.35619100
C	-3.36822700	-1.15117900	6.25806900
H	-3.21192400	-2.05639300	6.40112600
C	-5.66998600	-1.63725800	5.60753500
H	-5.48075100	-2.53979200	5.72768700
N	-9.71734000	1.70131100	3.06478800
C	-4.71290400	-0.68606500	5.92759500
C	-8.50473700	1.66280600	3.62404400
H	-7.85222700	2.32107700	3.53936000

C	-6.23300300	1.05415300	5.34468800
H	-6.43123800	1.96093500	5.28012600
C	-6.91039400	-1.24729500	5.10769500
H	-7.54936400	-1.88267300	4.88058000
C	-10.36616600	0.56955000	3.44029100
H	-11.22914500	0.33279300	3.17722400
C	-4.99717600	0.64961400	5.82903400
H	-4.36382500	1.28020900	6.08274100
C	-7.17234700	0.10725800	4.95998000
C	-9.55543300	-0.16622300	4.25985300
H	-9.76707500	-0.96434900	4.68264200
N	3.54540300	-7.40761400	4.34129000
N	1.11563600	-0.48072400	6.55305500
H	1.79252900	0.03820800	6.65741800
N	1.28319400	-1.82075100	6.35991600
C	2.48798500	-2.24024800	6.26319200
H	3.19484800	-1.65348500	6.40581000
C	4.06072500	-3.99192100	5.61616100
H	4.74756300	-3.37666700	5.73601800
N	3.19506700	-9.16982500	3.07970500
C	2.75821600	-3.63829100	5.93479900
C	2.62176000	-8.09960000	3.63708200
H	1.72541500	-7.86377300	3.55137900
C	2.01151400	-5.82564700	5.35419200
H	1.32551200	-6.45082800	5.28993600
C	4.34357500	-5.26178500	5.11824900
H	5.21353100	-5.49786800	4.89213300
C	4.49932600	-9.16525600	3.45622000
H	5.13603200	-9.79469200	3.19449500

C	1.74366800	-4.55245000	5.83661200
H	0.88067600	-4.31896100	6.08933400
C	3.30164600	-6.16614500	4.97094400
C	4.73057700	-8.09419700	4.27452300
H	5.52720000	-7.87784300	4.69764500
Pd	-10.42522100	3.10426500	1.80330900
N	-11.23583700	4.50132700	0.56305000
N	-10.99984000	4.33541400	3.33360800
C	-10.17017700	5.13156200	-0.30750500
H	-9.74427900	4.45374000	-0.83627000
H	-9.51502300	5.56031200	0.24891400
H	-10.57230700	5.78438000	-0.88201100
C	-11.87341700	5.53397000	1.40379100
H	-11.85498700	6.38316400	0.93632400
H	-12.80255100	5.29508100	1.55447700
C	-12.23807100	3.82564100	3.98907300
H	-12.92252600	3.70653500	3.32569400
H	-12.53320900	4.45666900	4.64696100
H	-12.05178900	2.98388700	4.41219600
C	-12.26034700	3.93963500	-0.28629500
H	-11.87193300	3.27045900	-0.85519100
H	-12.64415200	4.63303100	-0.82970800
H	-12.94346100	3.53947600	0.25605200
C	-11.22944800	5.67636500	2.64992600
H	-11.76784100	6.23881000	3.22755900
H	-10.37559100	6.11731600	2.51689400
C	-10.06284200	4.50637700	4.37746300
H	-9.91743600	3.66672400	4.81395100
H	-10.40056500	5.14391100	5.00861200

H	-9.23481700	4.82669400	4.01316400
Pd	2.33497400	-10.48605900	1.81932700
N	1.53142800	-11.88828500	0.58033300
N	1.55489500	-11.59713700	3.35052300
C	0.45344100	-11.28161300	-0.29188000
H	0.82790500	-10.57460400	-0.82130400
H	-0.24580700	-10.92795000	0.26351500
H	0.08957300	-11.95702800	-0.86576000
C	0.95517300	-12.95557300	1.42206100
H	0.21098700	-13.36478400	0.95456700
H	1.62661000	-13.64056800	1.57419100
C	2.61501500	-12.41372300	4.00791100
H	3.06083000	-12.94784400	3.34559600
H	2.21555200	-12.98392500	4.66625600
H	3.25049700	-11.83096800	4.43074400
C	2.53068600	-12.49583000	-0.26741900
H	2.91648500	-11.82551100	-0.83692200
H	2.12260300	-13.17559900	-0.81023300
H	3.21839100	-12.88651000	0.27599200
C	0.50890000	-12.46744200	2.66719800
H	0.29065900	-13.21409500	3.24566200
H	-0.29980300	-11.94863100	2.53283100
C	0.93760800	-10.86977100	4.39291400
H	1.59159900	-10.32348700	4.82917900
H	0.55378700	-11.48016100	5.02459000
H	0.24637400	-10.31332600	4.02732600
Pd	-0.38782300	10.86663400	-1.87286600
N	0.80967300	9.86352100	-3.12248400
N	0.76387700	2.11347000	-6.12201700

N	1.52371300	8.22833700	-4.43229400
C	2.83690800	6.31966200	-5.20491900
H	3.61781500	6.80705600	-5.07552700
N	0.97081300	0.76254300	-6.30803800
H	1.76021200	0.42998800	-6.38878700
N	0.60263700	2.15568200	2.95801000
C	2.50114900	9.21541200	-4.49027800
H	3.29254300	9.20559700	-4.97896000
C	0.52905800	8.64761900	-3.65955700
H	-0.25554200	8.16929100	-3.50425100
C	2.89626800	4.98388400	-5.56637100
H	3.72332700	4.56699100	-5.65274400
N	1.16579000	8.26338100	1.01101100
C	0.27022200	8.67085100	0.07723200
H	-0.44256900	8.15328500	-0.22119000
C	0.49343700	4.89835200	-5.69990600
H	-0.28487100	4.42732700	-5.89609800
N	0.87786900	0.85500200	3.24363500
H	1.68081600	0.59871300	3.41035600
N	0.52619600	9.87913300	-0.36401000
C	2.62686800	5.03668000	2.05275500
H	3.46294200	4.63360300	2.11848000
C	1.59298400	6.92443300	-5.03266200
C	1.82946400	2.82675200	-6.08811300
H	2.65788200	2.43351400	-6.24436400
N	-1.63331200	11.87274700	-0.65088600
C	1.74165100	4.27471600	-5.80105100
C	-2.69032600	12.55164000	-1.55951100
H	-3.38088800	11.91172800	-1.80006100

H	-3.10940800	13.28859700	-1.08866900
C	-0.11667700	-0.01221100	-6.31714000
C	0.42715300	6.23663200	-5.30595800
H	-0.39615700	6.66265500	-5.22538700
C	2.52101400	6.35145400	1.63259300
H	3.29183500	6.83465300	1.43280800
C	1.27342600	6.96227700	1.50678000
C	2.03308700	10.21614200	-3.65469000
H	2.48126000	11.01120100	-3.47669800
C	1.64623800	10.29011000	0.30887700
H	2.05456300	11.12356000	0.21542000
C	1.48752700	4.32639000	2.37485800
C	2.07290400	9.29778100	1.13715200
H	2.82819900	9.31071300	1.67603600
C	0.24327200	4.92319500	2.28651300
H	-0.51903500	4.44812300	2.52942400
C	0.13259000	6.26653900	1.81322500
H	-0.70215700	6.66492300	1.71897400
C	1.63184400	2.92625000	2.78138900
H	2.48367000	2.57842600	2.91576700
N	-1.28965700	11.90100400	-3.43553200
C	-2.53245800	10.97314500	0.21191800
H	-2.96516200	10.32239600	-0.34872600
H	-3.19582300	11.51013200	0.64716100
H	-1.99958700	10.52257300	0.87093800
C	-0.45806300	12.55366300	-4.26717400
H	0.07352600	11.91670500	-4.75137700
H	0.11377700	13.13237000	-3.76429200
H	-0.97259100	13.07557000	-4.89061200

C	-0.91339600	12.92248500	0.11890300
H	-0.39308700	13.45985900	-0.48153100
H	-0.33413400	12.50528100	0.76014400
H	-1.55223600	13.47672400	0.57636200
C	-2.01292300	13.03646800	-2.76040200
H	-1.37659600	13.73237900	-2.52002100
H	-2.66055400	13.41957900	-3.37507900
C	-2.19911500	10.95764700	-4.16541600
H	-2.76643100	10.50733600	-3.53395600
H	-1.67396200	10.31056100	-4.64081900
H	-2.73987300	11.45145500	-4.78445900
C	-0.12410300	-0.00793900	3.26187300
N	-9.13230800	-4.14256500	-3.12394000
N	-2.39530300	-0.31133600	-6.12378500
N	-8.07217700	-2.70839200	-4.43485800
C	-7.07523700	-0.61784900	-5.20952100
H	-7.88782100	-0.18507600	-5.08134700
N	-1.32816500	0.54339500	-6.30763000
H	-1.43599200	1.39276100	-6.39387700
C	-9.41566700	-2.35549200	-4.49436300
H	-9.80243700	-1.66586800	-4.98427400
C	-7.93856300	-3.77835600	-3.66057400
H	-7.13221900	-4.21846300	-3.50405200
C	-5.94776000	0.10100700	-5.57106700
H	-6.00020600	1.02560200	-5.65872100
C	-4.67215900	-1.93732200	-5.70086000
H	-3.87492800	-2.37612700	-5.89584600
C	-6.97716000	-1.99721900	-5.03532700
C	-3.54577900	0.25497500	-6.09115800

H	-3.61936100	1.16885700	-6.24865800
C	-4.75608100	-0.54464600	-5.80394200
C	-5.79835100	-2.66334900	-5.30680900
H	-5.75571300	-3.58925800	-5.22496000
C	-10.04890900	-3.26010000	-3.65805100
H	-10.96179400	-3.26925300	-3.48075700
N	7.96826400	-5.74951000	-3.10991500
N	1.28427200	-1.83483100	-6.12095400
N	6.19725400	-5.55028200	-4.42248600
C	3.88891000	-5.73329400	-5.19869700
H	3.92030100	-6.65318600	-5.06925900
N	0.00955400	-1.33726500	-6.30267500
H	-0.66996600	-1.85732600	-6.39520300
C	6.56344100	-6.89033600	-4.47989700
H	6.15995800	-7.57080300	-4.96920800
C	7.05648800	-4.89856000	-3.64840300
H	7.03426600	-3.97999900	-3.49313600
C	2.70294600	-5.11665500	-5.56199600
H	1.92852600	-5.62457500	-5.64957200
C	3.83048900	-2.99297200	-5.69377200
H	3.81200700	-2.08350500	-5.88999200
C	5.03436800	-4.95835800	-5.02465400
C	1.36897700	-3.11429900	-6.08583200
H	0.61446600	-3.63515400	-6.24318600
C	2.66643300	-3.76215200	-5.79672900
C	5.02202800	-3.60479500	-5.29797200
H	5.80250700	-3.10480000	-5.21618700
C	7.66279400	-6.98533200	-3.64259600
H	8.12697500	-7.77108200	-3.46388700

N	-2.35829300	-0.45980400	2.95690900
N	-7.92778500	-3.02850300	1.00899900
C	-7.83217200	-4.00912300	0.07661000
H	-7.02738600	-4.36797800	-0.22069900
N	-1.37006900	0.42905600	3.24292300
H	-1.54889800	1.25334500	3.40653900
N	-9.00614900	-4.39211200	-0.36503000
C	-5.86451400	-0.14878000	2.04721400
H	-5.93398400	0.77719600	2.11344400
C	-6.95011600	-0.89807900	1.62850500
H	-7.75380600	-0.47236200	1.42748900
C	-6.85515700	-2.28414300	1.50460000
C	-9.92263900	-3.62675800	0.30611400
H	-10.84856500	-3.69004000	0.21202300
C	-4.68072600	-0.77895400	2.37543200
C	-9.27725000	-2.75992600	1.13372700
H	-9.66654600	-2.11157600	1.67143800
C	-4.57471400	-2.15559300	2.28491700
H	-3.78260600	-2.57807400	2.53042300
C	-5.68254600	-2.92382300	1.81279800
H	-5.61003700	-3.84602800	1.71986900
C	-3.53968900	0.04592300	2.77677500
H	-3.66496600	0.95737600	2.91321700
N	1.38724100	-1.71626500	2.96065600
N	6.39808200	-5.25789500	1.02114600
C	7.20020600	-4.68600200	0.08861500
H	7.10881200	-3.80996800	-0.20995200
N	0.12259500	-1.30477800	3.24188000
H	-0.50081400	-1.87100900	3.41207400

N	8.11925900	-5.51178900	-0.35120300
C	2.87155900	-4.90943600	2.05566900
H	2.10450100	-5.43250800	2.12248200
C	4.06373600	-5.47557000	1.63912900
H	4.09707100	-6.38474300	1.43936000
C	5.21679700	-4.70053100	1.51522600
C	7.91412400	-6.68731400	0.32136500
H	8.43194200	-7.45760600	0.22871400
C	2.82628300	-3.57011700	2.38291300
C	6.84013400	-6.56070100	1.14797200
H	6.47277900	-7.22124200	1.68628400
C	3.96323900	-2.78701800	2.29196500
H	3.93413400	-1.89126100	2.53608200
C	5.18396500	-3.36475600	1.82106900
H	5.94661400	-2.84095400	1.72839100
C	1.54018300	-2.99219200	2.78428200
H	0.81310000	-3.55709100	2.91640400
Pd	-9.40320800	-5.67949000	-1.87246200
N	-9.65275100	-7.25951500	-0.64854700
C	-9.71149900	-8.51558900	-1.55553100
H	-8.81178800	-8.79404600	-1.79500600
H	-10.14050500	-9.24632300	-1.08403600
N	-9.84686100	-6.97978700	-3.43372600
C	-8.42473600	-7.58722200	0.21565300
H	-7.64440800	-7.63734000	-0.34431300
H	-8.55846400	-8.42963800	0.65193000
H	-8.30151800	-6.89957200	0.87384400
C	-10.82727700	-6.58702800	-4.26666300
H	-10.54107400	-5.80890800	-4.75169100

H	-11.61471500	-6.38048100	-3.76467100
H	-11.02151000	-7.29448900	-4.88929900
C	-10.92246100	-7.15988400	0.12012000
H	-11.64746000	-6.97888900	-0.48112500
H	-10.85123800	-6.44877800	0.76045700
H	-11.08329100	-7.98968000	0.57856600
C	-10.46915600	-8.17295400	-2.75747100
H	-11.39015500	-7.96961700	-2.51808100
H	-10.47662000	-8.92624100	-3.37114200
C	-8.57462700	-7.29667400	-4.16219300
H	-7.90143200	-7.56210200	-3.52985100
H	-8.27646900	-6.51909000	-4.63841000
H	-8.73144700	-8.01278100	-4.78039500
Pd	9.43384200	-5.21403400	-1.85801500
N	10.92601300	-4.63847500	-0.63370800
C	12.04389400	-4.06251900	-1.54060000
H	11.83531100	-3.14447100	-1.78147200
H	12.89090200	-4.06801100	-1.06843600
N	10.78299300	-4.95010800	-3.41858200
C	10.59513200	-3.41000600	0.22858100
H	10.24881500	-2.70989300	-0.33260200
H	11.39120800	-3.10399500	0.66506400
H	9.93740500	-3.64618500	0.88657700
C	10.93363400	-5.99672900	-4.24999500
H	10.11707000	-6.13856400	-4.73546900
H	11.14818100	-6.78122400	-3.74677800
H	11.64394600	-5.81202900	-4.87232600
C	11.47391000	-5.78679800	0.13693200
H	11.68019300	-6.50610100	-0.46318600

H	10.82193400	-6.07983300	0.77715400
H	12.27261600	-5.51063600	0.59563000
C	12.12685500	-4.89158400	-2.74136400
H	12.41105200	-5.79061800	-2.50054000
H	12.78347700	-4.52224500	-3.35501500
C	10.42189700	-3.69095300	-4.14903000
H	10.31464600	-2.97437400	-3.51773200
H	9.59968800	-3.82218600	-4.62571200
H	11.12090600	-3.46957700	-4.76698300
N	3.80389200	-0.12970100	4.40692400
O	3.73191700	0.98547200	3.91325500
O	2.65896400	-0.58464200	4.92578100
N	-1.79095900	-3.56400800	4.33521200
O	-1.32063800	-4.20820500	3.38603400
O	-0.95816500	-2.89752600	5.04611400
N	-2.27085100	3.40986200	4.14918800
O	-3.10195700	2.92877900	3.35364600
O	-1.59937800	2.57531700	4.84649700
N	3.86750900	0.46873600	-4.32519700
O	4.64164000	-0.17931900	-3.62057300
O	2.90624200	-0.17877900	-4.88617500
N	-1.07004900	-3.90144400	-4.24836200
O	-0.31650500	-2.91425900	-4.72383900
O	-2.11362100	-3.54316100	-3.71679700
N	-2.88652400	3.06285900	-4.01816800
O	-2.28404100	2.11043300	-4.62510100
O	-2.17855900	3.84005200	-3.35224400
O	-2.01467200	4.60202100	4.22119100
O	4.75273100	-0.87624600	4.45432800

O	-2.99213200	-3.44291900	4.55393900
O	-0.63001200	-5.02254600	-4.35100000
O	3.88733100	1.69241800	-4.45141600
O	-4.10723900	3.11373800	-4.04829600
C	6.03535100	1.57070400	-0.02071300
C	4.78569200	2.09788800	0.10120700
C	3.61702300	1.24833600	0.05085500
C	3.80164200	-0.17002000	-0.05672600
C	5.14387300	-0.68539200	-0.20736900
C	6.21710400	0.15262300	-0.19813700
C	2.31854100	1.77113700	0.09653300
C	2.68329500	-1.01275400	-0.02845100
C	1.38270100	-0.48975000	0.04580600
C	1.19618800	0.92784000	0.08322900
C	-0.15106600	1.45116100	0.10950300
H	-0.28442700	2.53327800	0.13656600
C	-1.22493500	0.61272000	0.10828900
C	-1.03668000	-0.81312800	0.09631900
C	0.21737300	-1.34487700	0.06353500
H	2.17402000	2.85085300	0.12469500
H	6.93013800	2.19540600	0.00421900
H	4.63311500	3.16395100	0.24103600
H	5.26577700	-1.75483900	-0.35420800
H	7.23707700	-0.21565000	-0.32910700
H	2.82265200	-2.09278500	-0.06230200
H	-2.24319000	1.00258300	0.11564600
H	-1.91959700	-1.45112400	0.12552200
H	0.37110300	-2.42364900	0.04466100

9. References:

1 C. Yu, B. Zhao, Y. Zhao and J. Lin, A new route for the synthesis of Ozagrel hydrochloride. *Org. Prep. Proced. Int.*, 2010, **42**, 183.

2 (a) T. M. McPhillips, S. E. McPhillips, H.-J. Chiu, A. E. Cohen, A. M. Deacon, P. J. Ellis, E. Garman, A. Gonzalez, N. K. Sauter and R. P. Phizackerley, Blu-Ice and the Distributed Control System: software for data acquisition and instrument control at macromolecular crystallography beamlines *J. Synchrotron Rad.*, 2002, **9**, 401. (b) N. P. Cowieson, D. Aragao, M. Clift, D. J. Ericsson, C. Gee, S. J. Harrop, N. Mudie, S. Panjikar, J. R. Price and A. Riboldi-Tunnicliffe, MX1: a bending-magnet crystallography beamline serving both chemical and macromolecular crystallography communities at the Australian Synchrotron *J. Synchrotron Rad.*, 2015, **22**, 187.

3 W. Kabsch, Automatic processing of rotation diffraction data from crystals of initially unknown symmetry and cell constants *J. Appl. Cryst.*, 1993, **26**, 795.

4 G. M. Sheldrick, Crystal structure refinement with SHELXL *Acta Cryst.*, 2015, **71**, 3.

5 O. V. Dolomanov, L. J. Bourhis, R. J. Gildea, J. A. Howard and H. Puschmann, OLEX2: a complete structure solution, refinement and analysis program *J. Appl. Cryst.*, 2009, **42**, 339.

6 (a) A. L. Spek, PLATON SQUEEZE: a tool for the calculation of the disordered solvent contribution to the calculated structure factors *Acta Cryst.*, 2015, **71**, 9, (b) P. van der Sluis and A. L. Spek, BYPASS: an effective method for the refinement of crystal structures containing disordered solvent regions. *Acta Cryst.*, 1990, **46**, 194.

7 (a) D. Yang, J. L. Greenfield, T. K. Ronson, L. K. S. von Krbek, L. Yu and J. R. Nitschke, *J.Am. Chem. Soc.*, 2020, **142**, 19856, (b) H. Takezawa, T. Murase, G. Resnati, P. Metrangolo and M. Fujita, *J. Am. Chem. Soc.*, 2014, **136**, 1786.

8 M. Frisch, G. Trucks, H. B. Schlegel, G. E. Scuseria, M. A. Robb, J. R. Cheeseman, G. Scalmani, V. Barone, B. Mennucci and G. Petersson, gaussian 09, Revision d. 01, Gaussian *Inc., Wallingford CT* 2009, **201**.
